# Imaging of Structural Timber Based on In Situ Radar and Ultrasonic Wave Measurements: A Review of the State-of-the-Art

**DOI:** 10.3390/s24092901

**Published:** 2024-05-01

**Authors:** Narges Pahnabi, Thomas Schumacher, Arijit Sinha

**Affiliations:** 1 Civil and Environmental Engineering, Portland State University, Portland, OR 97201, USA; thomas.schumacher@pdx.edu; 2 Wood Science and Engineering, Oregon State University, Corvallis, OR 97331, USA; arijit.sinha@oregonstate.edu

**Keywords:** structural timber, durability, ultrasonic testing, ground penetrating radar, imaging, synthetic aperture focusing technique, tomography, in situ examination, damage detection, condition evaluation

## Abstract

With the rapidly growing interest in using structural timber, a need exists to inspect and assess these structures using non-destructive testing (NDT). This review article summarizes NDT methods for wood inspection. After an overview of the most important NDT methods currently used, a detailed review of Ground Penetrating Radar (GPR) and Ultrasonic Testing (UST) is presented. These two techniques can be applied in situ and produce useful visual representations for quantitative assessments and damage detection. With its commercial availability and portability, GPR can help rapidly identify critical features such as moisture, voids, and metal connectors in wood structures. UST, which effectively detects deep cracks, delaminations, and variations in ultrasonic wave velocity related to moisture content, complements GPR’s capabilities. The non-destructive nature of both techniques preserves the structural integrity of timber, enabling thorough assessments without compromising integrity and durability. Techniques such as the Synthetic Aperture Focusing Technique (SAFT) and Total Focusing Method (TFM) allow for reconstructing images that an inspector can readily interpret for quantitative assessment. The development of new sensors, instruments, and analysis techniques has continued to improve the application of GPR and UST on wood. However, due to the hon-homogeneous anisotropic properties of this complex material, challenges remain to quantify defects and characterize inclusions reliably and accurately. By integrating advanced imaging algorithms that consider the material’s complex properties, combining measurements with simulations, and employing machine learning techniques, the implementation and application of GPR and UST imaging and damage detection for wood structures can be further advanced.

## 1. Introduction

Wood is a hierarchical and cellular material commonly utilized across various applications. Despite its widespread usage, the considerable variability in the properties and behavior of wood poses a significant challenge to gaining a comprehensive understanding of basic microstructure–property relationships. This variability, stemming from factors such as growth conditions, species differences, and aging, introduces complexity that hinders the establishment of consistent patterns in the relationships between wood’s microstructure and its properties. Thus, while the general concept of wood is well understood, navigating through the diverse and inconsistent nature of its characteristics remains a complex task [1]. Structural timber refers to wood used in various applications, such as walls, roofs, floors, bridges, etc. Wood is a versatile and durable material used for centuries to construct buildings and other structures. Its strength, sustainability, and cost-effectiveness make it a popular choice for engineers. Using timber as a building material has been a construction staple, but ensuring the safety and longevity of timber structures can be challenging. The life cycle of wood is over a hundred years if the wood is protected against environmental influences. There are several examples of wooden structures that have long life cycles, such as Stave Churches in Norway that were built in the 12th century, Kizhi Pogost in Russia that were built in the 18th century, Carpenters’ Gothic Churches in the United States that were built in the 19th century. Over time, timber can be susceptible to decay and damage that leads to strength degradation, compromising the integrity of a building or structure. For instance, the ‘big Wind River bridge’ in the USA was a timber bridge that collapsed in 2012 owing to decay in the wooden supports; a timber bridge in Norway collapsed in 2022 because of overload; and a pedestrian timber bridge collapsed in Finland because of unseen flaws. Therefore, developing robust NDT methods is essential to detect damage during the operational stage [2,3,4]. Imaging uses various techniques to visualize and characterize materials, components, structures, or systems. Imaging timber structures involves creating internal images of wooden structures using non-destructive testing (NDT) methods that do not damage the wood. This type of testing can help assess the quality of the wood and detect defects, decay, or cracks that may affect the structural integrity of the structural timber [5]. In recent years, new techniques for imaging timber have emerged, including ground penetrating radar (GPR) and ultrasonic testing (UST). The main reason for the emphasis on GPR and UST stems from their quantitative nature, allowing for the extraction of measurements that can be used to determine the location and quantify the extent of inclusions. Additionally, these techniques are deployable in situ, a capability not afforded by methods such as X-ray imaging. The two methods also present a safer and non-ionizing substitute for X-ray imaging, mitigating health risks for inspectors and minimizing environmental impacts. In contrast to the conventional drilling resistance method, which introduces holes into the material, GPR and UST distinguish themselves as entirely non-destructive, thus fully preserving the structure’s integrity. Both techniques use waves to penetrate and interact with the timber. They produce measurements that can be translated into an image, representing a depth slice for straightforward interpretation and extraction of geometric information. While sounding provides a qualitative map, the images generated by GPR and UST can be reconstructed using identical algorithms (such as SAFT and TFM) and prove to be mutually complementary [6].

In this paper, we aim to provide a comprehensive overview of imaging methods, both those already in use and those with the potential for application in NDT, including state-of-the-art in situ radar and ultrasonic wave measurements for imaging timber. We will discuss their benefits, limitations, and potential applications in engineering. Section 2 describes the structure of wood, how wood is manufactured into timber products, and properties of wood and timber. Section 3 summarizes NDT techniques applied to wood and timber and how they are implemented. Section 4 outlines the definition of imaging, the state-of-the-art in imaging, fundamentals of imaging, B-scan, SAFT, and tomography; GPR and UST-based imaging, and the differences between imaging techniques and tomography are discussed. The last section presents conclusions and an outlook on future research.

## 2. Properties of Wood and Timber

**Figure 1:**
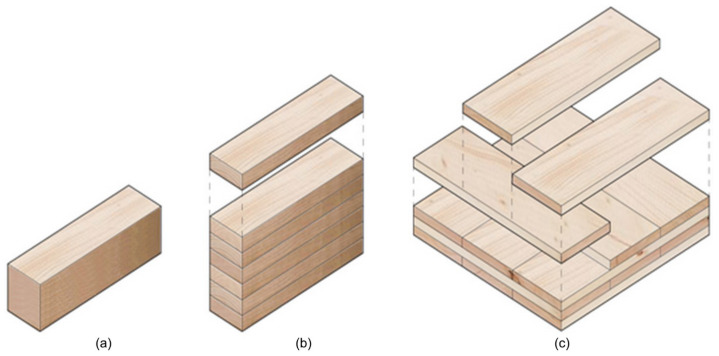
Examples of timber and mass timber systems. (**a**) Simple timber, (**b**) glued-laminated timber (Glulam) beam, (**c**) cross-laminated timber (CLT) slab. Adapted from: [15].

Wood is a biomaterial composed of cellulose (up to 45%), lignin (25–30%), hemicellulose (20–30%), and minor amounts (usually less than 10%) of extraneous materials contained in a cellular structure. Variations in the characteristics and proportions of these components and differences in the cellular structure make woods heavy or light, stiff or flexible, and hard or soft. Unlike many materials used in construction, wood is a renewable resource and offers a sustainable option. Wood boasts a high strength-to-weight ratio. This translates to lighter structures that require less foundation support, reducing overall construction costs. It has natural insulation properties, offering excellent thermal and acoustic insulation, contributing to energy efficiency and occupant comfort in buildings. It is generally easier to work with and shape than other materials [7,8]. Wood’s electrical conductivity, dielectric properties, and mechanical characteristics vary with moisture content, grain direction, and loading orientation, presenting challenges for NDT. Its porous nature adds complexity to managing shrinkage, swelling, and hygroscopicity. The anisotropic properties demand precise NDT techniques to avoid misinterpretations. Wood’s susceptibility to biodegradation requires assessing degradation without compromising structural integrity. These diverse properties collectively pose challenges for achieving consistent and accurate non-destructive evaluations of wood structures, necessitating careful calibration [9]. Timber refers to wood processed and cut into lumber or other usable forms. Depending on the species, region, growth conditions, and processing techniques, it exhibits various mechanical and electrical properties. Mass timber (MT) is a group of large engineered structural wood panels such as cross-laminated timber (CLT), sometimes termed x-lam or cross-lam, glue laminated timber (Glulam), laminated veneer lumber (LVL), and so on. Due to its sustainable construction, it has been widely popular. It uses state-of-the-art technology to glue, nail, or dowel wood products together in layers. The outcomes are large structural panels, posts, and beams. These products are thick, compressed layers of wood, creating strong, structural load-bearing elements that can be constructed into panelized components. They are usually made through lamination, fasteners, or adhesives. The benefits of MT-based buildings would be their low carbon emission, short construction time, and cost-effectiveness. However, their durability could be of great concern [10,11,12,13,14]. Figure 1 shows schematics of different types of mass timber.

Macrostructure and microstructure further distinguish wood from other materials. The concentric ring structure, resulting from uneven growth rates, introduces local strength and stiffness variations, impacting crack growth patterns during stress. Microscopic features, such as cell size, arrangement, and composition, contribute to the anisotropic behavior of wood, presenting challenges in NDT techniques reliant on consistent material properties. These features are discussed below.

### 2.1. Macro and Micro Structure

**Figure 2:**
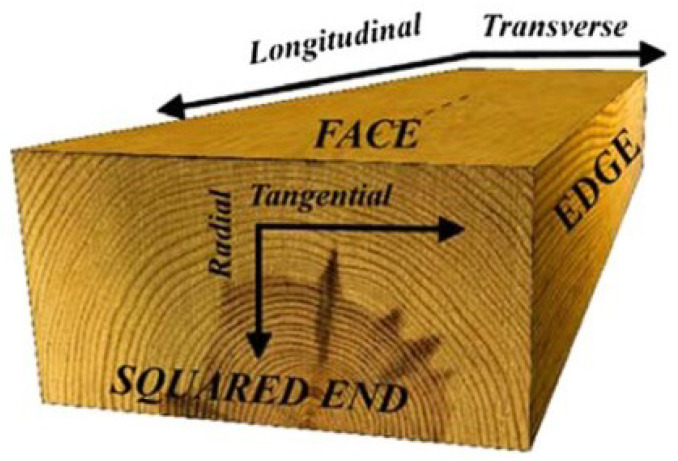
Principal axes of wood concerning grain direction, growth rings, and coordinate system: longitudinal (*L*), transverse (⊥), tangential (*T*), radial (*R*). Source: [16].

Macrostructure in wood refers to the visible characteristics of the wood at a larger scale that is visible to the naked eye, including the size, shape, grain pattern, growth rings, knots, the orientation of wood fibers, texture, and other surface characteristics. Microstructure, on the other hand, refers to the cellular structure of the wood at a smaller scale that requires optical tools. These features include the size and arrangement of individual cells, cell wall composition and distribution, and various pores, canals, and other structural features. Wood’s microstructure and macrostructure are important in determining its mechanical and electrical properties. These features can provide important information about the properties of wood, including its strength, stiffness, durability, and resistance to decay [8]. The concentric ring structure in a tree log results from the tree’s uneven growth rate throughout the year. Earlywood, formed during the rapid growth period of spring and early summer, is characterized by large-diameter cells. In contrast, latewood, developed in late summer and autumn, consists of smaller cells. The transitions between earlywood and latewood are crucial in creating a continuous variation in local strength and stiffness. The boundary between latewood and earlywood tends to act as a cleavage plane, serving as a preferred path for crack growth under specific stress conditions. Figure 2 illustrates the standard coordinate system used to describe the structure formed by these growth rings.

Wood material exhibits distinct responses to stresses applied along the fiber axis compared to those oriented across the fiber axis. The highly porous nature of wood, with typical porosity ranging between 50–60%, is attributed to its cellular structure. Consequently, the structural performance of wood is intricately linked to the properties of the fibers, their connections, and the interactions between them. The performance of wood is intricately linked to its fibers’ properties, connections, and interactions. However, one of its drawbacks is the notable shrinkage and swelling in the direction perpendicular to its fibers when exposed to water. Before the transformation of wood into timber products, the hollow cells are typically saturated or nearly saturated in the green state, either in a living tree or shortly after being felled. Moisture exists in the cell lumen (free water) and the cell wall (bound water). During the drying process, the free water in the cell cavity is the first to be removed, followed by the bound water. Only bound water remains when the wood is sawn and utilized [1].

### 2.2. Electrical Properties

The electrical properties of wood specifically pertain to how wood behaves in the presence of electric fields. These properties are relevant to electromagnetic waves (such as those used for GPR) and electrical measurements, consisting of electrical resistivity, conductivity, dielectric properties, anisotropy of wood, etc. All these electrical properties of wood are affected by moisture content, temperature, the applied frequency; wood species, wood density, grain orientation, thickness, chemical composition, cellulose, lignin, hemicelluloses, presence of cavities, discontinuities, and preservative treatment. They influence the electromagnetic field attenuation, phase shift, and polarization [8,17].

**Electrical resistivity:** The electrical resistivity of wood is a measure of its intrinsic resistance to the flow of electric current. The resistivity of wood can vary depending on factors such as wood and timber species, density, moisture content, grain orientation, and temperature. It is many orders of magnitude higher than metals and conductive materials. Higher resistivity means that wood offers more resistance to the passage of electric current. The density of wood and timber can affect resistivity, with denser woods typically having lower resistivity. Moisture content is one of the primary factors affecting the electrical resistivity of wood. As moisture content increases, the resistivity decreases. Water acts as a conductor, allowing the flow of electric current through the wood. Also, along the grain (longitudinal direction), wood generally exhibits lower resistivity than across the grain (transverse direction). In general, resistivity tends to increase with temperature; however, the temperature dependence in wood can vary depending on factors such as species, moisture content, and other physical properties [18].

**Electrical conductivity:** Electrical conductivity is the inverse of electrical resistance. Hence, wood is considered a poor conductor of electricity compared to conductive materials such as metals. The low conductivity is mainly attributed to the insulating properties of the wood’s cellular structure and the limited mobility of electrons within the material. Dry wood has higher resistivity and lower conductivity than wet or moist wood. This is primarily due to free ions and increased water content in the cell walls, which enhance the conductivity. The electrical conductivity can vary significantly with the direction of current flow relative to the wood’s grain direction. Wood conductivity is several times higher along the grain compared to across the grain [19].

**Dielectric properties:** dielectric properties describe a material’s response to an electric field, including permittivity and loss tangent. Permittivity, also known as the dielectric constant or relative permittivity, quantifies the ability of wood to store electrical energy when subjected to an electric field. Relative permittivity is the ratio of permittivity of a material to the permittivity of space or vacuum. The loss tangent represents the energy loss in the material due to the applied electric field. The presence of water heavily increases the relative permittivity of wood [16]. Increasing moisture content thus increases its permittivity, and the permittivity of different wood species is different; for instance, the permittivity of Spruce wood is higher than that of Pinewood, both in the parallel direction. Also, the relative dielectric permittivity is higher in the direction perpendicular to the fiber compared to the parallel direction [7]. Based on research on various species of timber with moisture content, it is reported that the dielectric constant of the longitudinal direction is higher than that of the transverse direction. Also, the amplitude in the parallel direction to the fiber is higher than the propagation in the perpendicular direction. Different wood densities can affect the dielectric constant. Generally, higher-density woods have higher dielectric constants due to the increased number of atoms and molecules available to interact with the electric field [2,20,21]. Table 1 provides the dielectric constant of different wood species at different moisture contents and longitudinal and transverse directions at 2.45 and 2.7 GHz frequencies.

The relationship between the velocity of the wave in wood (C) and the dielectric constant of that wood relative to a vacuum ($\epsilon_{r}$) is described by the formula [22]:

(1) $$C=\frac{c_{0}}{\sqrt{\epsilon_{r}}}$$

where $c_{0}$ represents the speed of light in a vacuum.

**Anisotropy:** Wood is an anisotropic material with different electrical properties along different directions. This anisotropy arises from the arrangement and orientation of wood fibers, vessels, and other cellular components [2,7,16]. The anisotropy of wood can indeed cause the speed of electromagnetic wave propagation to increase parallel to the grain direction and decrease in the other directions (perpendicular to the grain).

The above-mentioned factors influence the behavior of electromagnetic waves, particularly in the context of GPR. Moisture content is a major factor. Wet wood conducts electricity better, slowing down GPR waves and attenuating them. On the other hand, propagation at low moisture content is faster and has a higher amplitude. Wood density also matters. Denser wood generally allows GPR waves to travel farther with less attenuation [23,24].

### 2.3. Mechanical Properties

Wood and timber have various mechanical properties defining their behavior under mechanical waves. Here are some important mechanical properties of wood and timber [25]:

**Strength:** It refers to the ability of a material to withstand applied forces without failure. It is typically characterized by tensile, compressive, shear, and bending strength. The strength of wood varies depending on factors such as species, density, moisture content, and grain orientation. The strength of wood is highest along the grain and weakest across the grain. Hence, it allows the ultrasonic wave to travel faster with less interference along the grain [26].

**Stiffness:** Modulus of elasticity or Young’s modulus provides a measure of stiffness, representing the resistance of a material to deformation when subjected to an applied force. It indicates how much the wood will deflect when a load is applied. Stiffness influences the structural integrity and performance of wood in load-bearing applications. Higher stiffness typically leads to a higher natural vibration frequency in wave-based methods. Longitudinal stiffness is usually higher than radial or tangential stiffness in wood [27].

**Hardness:** It refers to the ability of wood to resist indentation or scratching. Wood species, density, and chemical composition influence this property.

**Durability:** This is the ability of wood to withstand environmental conditions and resist decay, rot, pests, and other forms of deterioration. When acted upon by biological agents of degradation, the wood exhibits mass loss, implying a loss in density. It is important for outdoor applications or structures exposed to moisture and biological agents. Wood species differ in their natural resistance to decay, and additional treatments or preservatives can enhance durability [14].

Mechanical waves, like ultrasonic waves, require a medium (solid, liquid, or gas) to propagate. They transmit energy through the vibration or oscillation of particles within the medium. They can be longitudinal (compression) waves, transverse (shear) waves, or surface (Rayleigh) waves. Properties of wood and timber governing mechanical wave propagation, such as wave velocity, include modulus of elasticity, fiber direction, density, moisture content, temperature, and presence of defects. Wood interacts with sound in different ways that captivate, produce, and intensify acoustic signals [27]. The anisotropy of wood results in the bending of wave paths and interferes with the propagation of ultrasonic waves by causing them to travel at different velocities in different directions [28].

Discontinuities in wood can lead to weaknesses and reductions in mechanical strength. Also, when a stress wave encounters a knot, void, or crack, its energy may be partially reflected or refracted, causing it to travel a longer path [23]. Knots can distort fiber orientation and discontinuities and introduce a local heterogeneity, where their properties are distinguishable from other areas. For example, they may have a higher density (if not rotten), which results in more wave attenuation. In addition, they can cause high-velocity values in ultrasonic imaging [18]. Insect attacks can cause voids and reduce wood density [29]. Fungi decay can be related to low-velocity areas in ultrasound imaging because of low material density [18]. Also, an increase in temperature range results in a decrease in wave velocity. Preservative treatment can somehow affect the velocity of stress waves. While timber treatment with waterborne salts may not affect stress wave velocity, the treatment with oil-borne preservatives reduces it [27].

Some properties of different species of sound wood are shown in Table 2.

**Figure 3:**
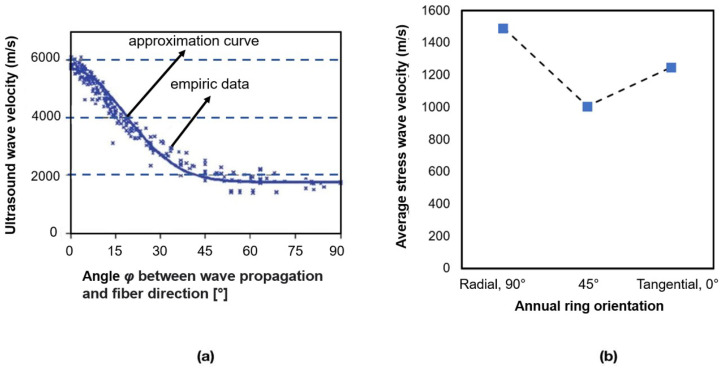
(**a**) Relationships between the velocity of the ultrasound compression wave and the angle between propagation and fiber direction in European spruce lumber (moisture content not available) (Source: [32]), (**b**) average compression wave velocity in good-quality wood with the moisture content of 12% is a function of annual ring orientation (figure based on data from [30]).

Wood density plays a significant role in the propagation of mechanical waves. It determines the mass per unit volume of the wood material. Different wood species have different densities, affecting wave propagation speed. For instance, higher mass concentration typically leads to a lower wave speed, and hardwood species are higher than softwood species. The equations for longitudinal (compression or P-wave) and transverse (shear or S-wave) waves are shown below:

(2) $$\begin{array}{cc} V_{p} & =\sqrt{\frac{k+\frac{4}{3}\mu }{\rho }}\;\\ V_{s} & =\sqrt{\frac{\mu }{\rho }} \end{array}$$

with, *k* being the bulk modulus, μ the shear modulus, ρ the density of the material. Wave velocities and wave behavior vary depending on the direction of wave propagation relative to the wood’s grain direction. When the ultrasonic wave travels in the direction of the grain, it encounters a more rigid and uniform path. This allows the wave to propagate faster with less interference [31]. A higher modulus of elasticity allows mechanical waves to travel faster through the material. Figure 3a shows the relation between the compression wave velocity in wood and the wave propagation angle and fiber direction. Also, Figure 3b shows the relation of compression stress wave velocity with annual ring orientation.

**Figure 4:**
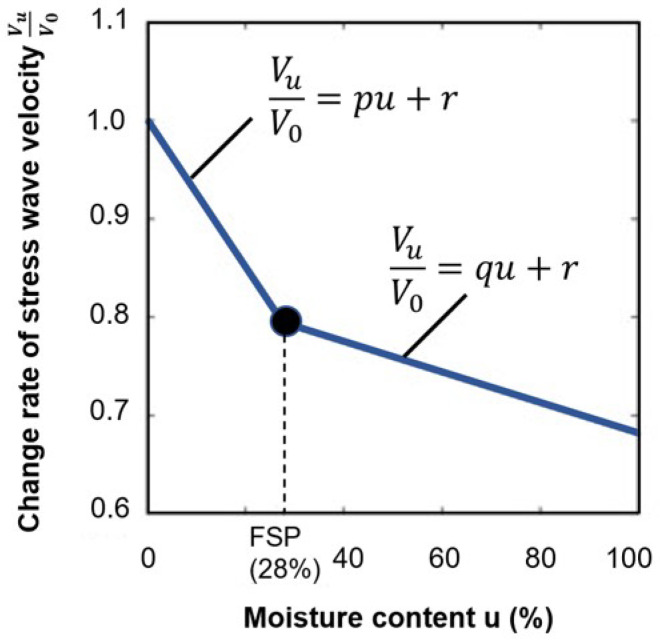
The relationship between wood moisture content and compression wave velocity. Source: [33].

It can be seen that as the angle increases, the ultrasound wave velocity generally decreases. Also, increasing the moisture content to the fiber saturation point will decrease the velocity of ultrasound waves. Figure 4 illustrates the correlation between relative compression wave velocity change and moisture content.

Acoustic impedance is a property that describes the resistance of a material to the transmission of sound waves. When an ultrasonic wave encounters an interface between two materials with different acoustic impedances, some wave energy is reflected into the first material while the remaining energy is transmitted into the second. The magnitude of reflection and transmission depends on the difference in acoustic impedance between the two materials. The acoustic impedance of a material is calculated by multiplying its density by the speed of sound in that material, as follows:

(3) $$\begin{array}{cc} Z & =\rho \times V\; \end{array}$$

where *Z* is acoustic impedance, ρ is density, and *V* is the speed of sound [34]. If the acoustic impedance of two materials is similar, there is less reflection at the interface, and more energy is transmitted. However, if there is a significant mismatch in acoustic impedance, a greater proportion of the wave is reflected, resulting in reduced transmission. In ultrasonic testing or imaging, this acoustic impedance mismatch enables the detection of interfaces, boundaries, or defects within the wood. Analyzing the reflected waves makes it possible to obtain information about the material’s internal structure, including determining the presence of flaws and measuring thickness. It is important to note that the acoustic impedance mismatch is not the only factor affecting the reflection of ultrasonic waves. Other factors, such as the angle of incidence, the frequency of the waves, and surface geometry and condition, also affect reflection behavior [35].

## 3. Non-Destructive Testing

Non-destructive testing (NDT) enables the assessment of a material, component, structure, or system for defects, anomalies, or irregularities without causing harm to the structure or impairing its functionality. NDT can help ensure structural integrity, reliability, and failure prevention. Without it, the safety and reliability of components can be seriously threatened over time [36,37]. Any decay that changes the wood’s electrical and mechanical properties and that may not be exposed on the surface can be detected using NDT [2]. Using assessment techniques on structural timber can provide valuable information about its condition, quality, and suitability for use in construction; this can help ensure structures’ safety and durability. Some techniques that have been applied to structural timber are described next.

**Visual Inspection:** The first and most basic technique is visual inspection, allowing the detection of external wood decay, moisture stains on exposed surfaces, visible mechanical damage, and defects. However, identifying subsurface damage is often not possible. Hence, more advanced NDT techniques should be considered to complement visual information [9].

**Sounding:** The original form of stress wave-base testing is considered one of the traditional methods used to examine in situ timber members. This method is simple to operate, and the inspection procedure can be carried out quickly to detect severe decay in timber. In this method, a timber member is struck with a blunt object, typically a hammer. The impact generates vibrations that manifest as sound energy propagating through the air through compression waves. The human ear’s subjective response to the frequency content of these compression waves is referred to as the sound’s pitch. The inspector can assess the condition of the timber based on the resulting sound tone, drawing on their experience. A sound wood pole produces a solid sound with a sharp hammer rebound (high-pitched sound). Conversely, a wood pole with internal deterioration generates a hollow or dull sound with a less pronounced hammer rebound (low-pitched sound) [38].

Although the method can rapidly screen timber members, it is subjective, and the results depend on the inspectors’ experience. Also, the sound quality may be affected by factors other than decay, which complicates the interpretation. The extent of damage or deterioration cannot be quantified, and while experienced inspectors can observe severe decay, initial and intermediate degree of decay are difficult to detect using the sounding technique [9,39,40,41].

**Infrared Thermography (IRT):** IRT produces images from infrared thermal radiation emitted by the surface of the tested member. The final image is displayed in the visual range using a predefined colormap. This technique can be carried out remotely in active and passive modes. In active IRT, there is a need to produce energy stimulus artificially to initiate internal heat flow in the tested member; this can be achieved by different processes such as lamps, heaters, flashes, etc. In contrast, natural environmental temperature fluctuations are used in passive IRT. The analysis of this technique can be qualitative and quantitative, whereby the qualitative part focuses on the thermal patterns to identify the existence and locations of damage; the quantitative part is related to determining the damage severity. IRT is performed utilizing infrared cameras. The resulting digital thermographic images are processed and analyzed using digital image processing software such as FLIR thermal studio suite, testo IRSoft, and so on [42,43,44,45].

Pitarma et al. utilized IRT to detect wood damage. This research used active IRT to apply energy on the wood surface. The latter could be identified through temperature variations, which result in different radiation patterns between intact regions and regions with defects. However, technical improvements were needed to achieve better resolution. The authors mention that high-resolution cameras supported by image processing software features must analyze the results better [42]. Kucharska and Jaskowska-Lemanska investigated using active IRT to detect knot area ratios in painted timber elements. They employed three external energy sources: a laboratory dryer, an air heater, and halogen lamps, along with a FLIR thermal imaging camera. Results indicated the method’s ability to estimate knot positions due to temperature variations, with the air heater being the most effective source. However, limitations include the need for access to all sides of the wood element and the subjective measurement of knot diameter in thermal images [46]. Gallego et al. used the IRT technique to inspect the width, length, and depth of superficial cracks in wooden beams. Two different methodologies were utilized: thermal images obtained without previous thermal excitation on the wooden beams and the image sequence obtained during the cooling after a thermal excitation in each wooden specimen. The outcomes illustrated that although the length and width of the cracks could be measured utilizing the thermal images of the wooden beams without previous thermal excitation, low-depth cracks had the same temperature gradient as high-depth cracks. So, the first methodology was not reliable enough. On the other hand, using the second methodology produced better results. Also, it was concluded that the combined use of the two modes was satisfactory; however, there was a need to use algorithms to automate the proposed methodologies [47]. Martinez and Martinez focused on passive IRT and imaging to assess the condition of historic structural timber. Also, this technique’s limitations and most frequent errors were identified to avoid misleading interpretations. The surface temperature measurement in passive IRT was carried out directly. The results showed that passive IRT detects only damage that generates camera-detectable thermal variations. While active IRT is not applicable in on-site situations, it was suggested that it can identify internal defects. IRT was also effective for rapidly assessing large surfaces, but it could be combined with other techniques, such as ultrasonic pulse velocity, to produce better results. Environmental conditions highly influenced the results [48].

**Figure 5:**
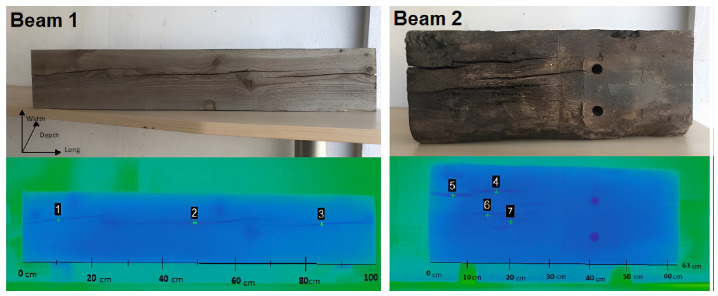
Thermal imaging of Beams 1 and 2 before any thermal excitation, revealing the presence of seven cracks. Source: [47].

Figure 5 shows the use of the IRT method in finding cracks in wooden beams. Seven cracks were analyzed, three for a pine beam (Beam 1) and four for an oak beam (Beam 2). The cracks are visible in the thermal images due to a thermal difference between these defects and the unaltered surroundings of the beams.

Regarding the methodology, while IRT appears promising, certain details and enhancements need consideration. Active IRT becomes indispensable when a sufficiently robust passive source is unavailable. It facilitates the differentiation of various wood types based on density differences. This confirms the influence of density on surface temperature. Perceiving the surface structure of wood becomes possible through IRT, making it valuable for evaluating the quality of the wood finish. Detecting knots and other surface defects is possible because of the density changes between these imperfections and the surrounding (undamaged) wood. Distinct moisture content zones within a piece of wood can also be identified through IRT and are attributed to thermal properties like specific heat, conductivity, and diffusivity alterations. However, passive IRT cannot detect internal wood deterioration when its moisture content matches that of the surrounding sound wood, regardless of size and depth beneath the surface. In contrast, active IRT excels in detecting internal defects with the same moisture content. Moreover, active IRT effectively identifies internal defects with higher moisture content than sound wood. Nevertheless, small internal defects with the same moisture content as the unaffected wood remain undetectable through IRT, even when an external stimulation source is applied [42,49].

**X-Ray Imaging (XRI):** XRI is one of the techniques used on structural timber and works without any contact with the member. Radiographic images are produced based on the intensity of radiation exposure on an imaging plate. An XRI system comprises an X-ray generator, a digital imaging system, and a reusable phosphor-layered imaging plate that captures the intensity levels of the specimens exposed to the X-rays. The emitted X-rays appear on images as lighter or darker in the scanned images because of the intensity loss through the specimens [9,39,50].

**Figure 6:**
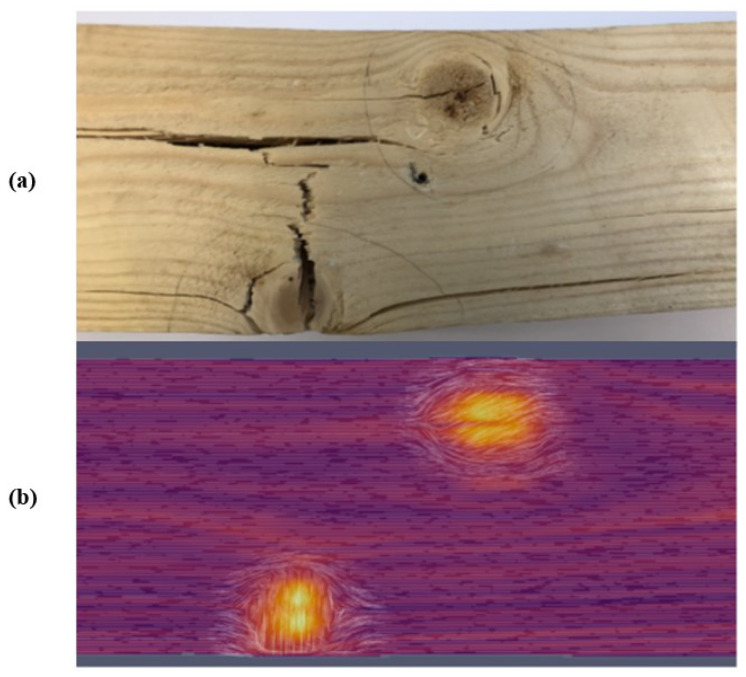
(**a**) The board was under test, with initial failure near the bottom-left knot, and (**b**) X-ray CT reconstruction at a depth of 10 mm. Source: [56].

Lechner et al. evaluated the density of structural timber components utilizing X-ray equipment that radiates short-wave electromagnetic rays. Some image configurations were applied to specimens, and an image toolbox was used to analyze and evaluate the produced images. A calibration wedge was set up to verify the procedure. Based on the outcomes, the technique had good potential to estimate the density of structural timber components. The image quality was high, making the radiograph interpretation straightforward [50]. Franke et al. demonstrated the capability of mobile X-ray technology for in situ assessment of structural timber members. Looking inside the member with high resolution had become feasible by using this technique. The use of mobile X-ray technology in combination with the specific digital scanner made it possible to assess the existing structures and led to excellent results. The results demonstrated the potential for assessing timber structures in laboratory tests and practical situations. The knots and even the annual rings were visible in the selected specimens. However, some timber structures experts should analyze the resulting radiograms reliably to detect irregularities from inaccuracies even in low-contrast radiograms [51]. Ge et al. used X-ray computed tomography (CT) to explore wood interiors non-destructively. Sinusoidal and sectional images were reconstructed with scanning data. Since CT is a medical instrument, a CT tomography test platform was developed to expand the application of CT technology in the NDT for wood. Although the outcome 3-D images showed a large amount of information about the properties of wood specimens, the software function of the system was not very powerful. Hence, there was a need for the image processing and analysis function of the self-developed CT software system to be improved [52]. Mckinley et al. investigated the penetration of an iodinated phenol-formaldehyde adhesive into Douglas-fir cell lumens and walls using advanced imaging techniques like nano-XCT. A gradient of penetration within the cell wall structure was observed. The conclusion highlighted the challenges faced during nano-XCT scans, successful scans achieved by pretreating samples, and evidence of adhesive penetration into the cell wall at a depth of 0.5–1 µm. The study suggested potential improvements in sample preparation, scanning conditions, and image reconstruction for future nano-XCT studies [53]. Paris et al. utilized micro-scale X-ray computed tomography (XCT) for detecting 3D adhesive distribution in wood-adhesive bond lines. There was a need for sufficient gray-scale contrast in the reconstructed XCT data to visualize and segment the material of the adhesives from the surrounding cellular structures. The effect of phase contrast at material boundaries could complicate image quality and segmentation in XCT data reconstructed with conventional algorithms. Hence, a quantitative phase retrieval algorithm was represented to isolate and remove the phase-contrast effect. It turned out that although X-ray CT was an applicable method, the segmentation of adhesive from cell walls in wood was complicated. However, phase retrieval algorithms could identify material contrast by eliminating phase-contrast effects [54]. Hwang et al. suggested synchrotron X-ray microtomography (SR-MCT) as an efficient method for imaging the wood’s internal microstructure. The microstructure of wood samples that optical methods could not visualize was reconstructed into three-dimensional images. This reconstructed image resolution was sufficient to perceive the main anatomical wood features. SR-MCT was considered an effective method that could even be used for historical buildings, which are of great importance [55]. Figure 6 illustrates the X-ray image of a wooden specimen. As can be seen, knots are visible.

XRI provides a clear and detailed image of the internal structure of wood, allowing for the detection of even small defects like cracks, knots, and insect damage. They can penetrate deeper into wood than NDT techniques like ultrasonic testing (UST). This allows for internal inspection of thicker pieces of wood, making it useful for assessing structural timber. X-ray images can provide quantitative information about wood properties, such as density and moisture content. However, its equipment is expensive to purchase and maintain. It is less sensitive to certain defects like fungal decay or early stages of insect infestation. These might require additional techniques like visual inspection or moisture meters for confirmation. X-ray imaging is typically conducted in controlled laboratory environments due to the potential health risks from radiation exposure and to ensure accurate results. This can limit its usefulness for field applications where on-site inspection is required.

**Electrical Resistivity (ER):** This method is often referred to as the impedance method and is used to determine the moisture content in wood; its application for internal inspection is relatively new. Two electrodes are inserted into the timber, and electrical resistivity is measured. The data are converted into moisture content (MC%) using empirical relationships. Additionally, further insights into the wood’s condition can be gained by employing a ring array of needle electrodes. Pairs of electrodes are utilized for current injection, while others are used for voltage measurement [57]. Variations in the placement of electrode pairs enable an interior property scan, which, in turn, allows the generation and assessment of a tomogram based on the collected data. ER data also enable the evaluation of both amplitude and phase, offering complementary insights into potential decay and the extent of the damage. This technique exhibits sensitivity to MC, electrolyte content, and anisotropy. It is influenced by various factors, including the type and age of the tree, as well as the year’s season.

**Figure 7:**
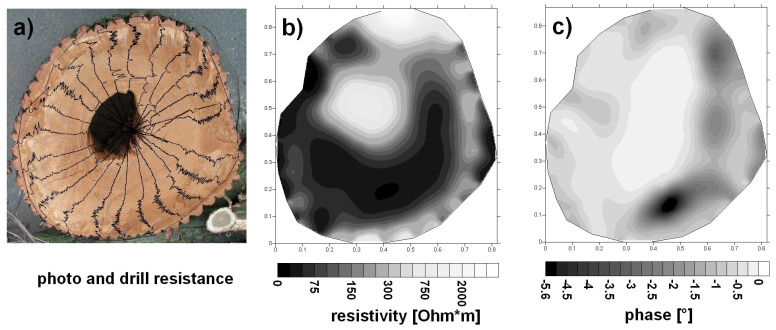
Illustrative findings from a fungus-infected tree: (**a**) Drill resistance results for comparison, (**b**) Electrical resistivity tomograms of sampled areas, and (**c**) Corresponding phase images from (**b**). Source: [61].

Dung et al. presented a new approach for monitoring MC in timber structures using electrical resistivity tomography (ERT). The method mapped timber resistivity about moisture content by analyzing current lines generated by electrodes connected to the timber surfaces. It utilized a numerical model of electrical current injection in finite element software called Castem, employing an error-minimization approach to match simulations with actual measurements. Subsequently, an inversion algorithm reconstructed the timber resistivity field based on the collected data, offering a comprehensive approach to moisture assessment in structural timber [58]. Soge et al. utilized the four-point electrical resistivity method, deployed in the field and laboratory, to detect the location and extent of wood decay and hollows in living acacia trees. Healthy trees exhibited increasing resistivity values from the perimeter to the center, while decayed trees had resistivity values five times lower, and hollowed trees had values four times higher. Laboratory experiments matched field results, providing a benchmark for identifying decay and hollows in living trees. However, fungi activity can lower resistivity without causing decay, limiting its use. Seasonal and moisture variations also affect resistivity measurements, especially for absolute values [59]. Hwang et al. used various electrodes to measure the MC of wood through electrical resistance and resistivity. They employed the two-pin method, conductive fabrics, and multi-pin electrodes for resistance measurements in Japanese larch wood, while a four-pin probe was used for resistivity. The two-pin method was less sensitive to wood sample dimensions, while the others were influenced by contact area and length between electrodes. A regression model using two-pin resistance data accurately predicted MC. The four-pin probe consistently provided stable resistivity values, making it a reliable method for assessing moisture in large wood components [60]. Figure 7 shows the results of using the ERT on a fungi-infected oak with a heart rot in the darker part. The gray-scale image indicates the tree’s electrical resistivity distribution, where the damaged areas appear brighter, i.e., have higher resistivity than the surrounding sound wood.

As depicted in Figure 7a, a central hole is present in the tree. The wood surrounding the hole is infected and has already reached a brittle state. The hole creates a region with notably high resistivities (see Figure 7b) and extremely low phases (refer to Figure 7c). The fungi continue progressing near the hole, resulting in relatively low resistivities and phases. Healthy wood sections exhibit moderate resistivities and high phases. The drill resistance measurements also highlight a substantially weakened zone at the tree’s center. Generally, these drill resistance findings align with those obtained through CRT. However, while drill resistance indicates robust wood on the left border, electrical resistivity, and phase measurements suggest the fungal infection has already affected this border.

The ER method can detect internal defects. However, generating and interpreting the tomograms demands experience, as moisture or electrolyte content alterations may not always correlate with decay. The wood’s resistivity is heavily influenced by its moisture level. This makes it challenging to differentiate between changes in resistivity caused by defects like decay and those caused by natural moisture variations, leading to inaccurate defect detection. The need to consider moisture content and anisotropy during data analysis increases the complexity and potential for misinterpretation, decreasing the method’s effectiveness compared to other NDT techniques [39].

**Ultrasonic Testing (UST):** UST uses high-frequency stress waves to detect internal flaws, measure thickness, and determine material characteristics [44,62]. The frequency range is between 20 to 500 KHz, and this amount should be kept low because wood is heterogeneous, and high waves will be attenuated [63]. The measurement setup consists of a signal generator, a preamplifier, an amplifier, transducers, and a recorder that collects and stores information that should be further analyzed. Two types of setups can be used for scanning: (1) contact-based scanning and (2) non-contact-based scanning. In contact-based scanning, transducers are coupled to the material surface directly to transmit ultrasonic waves into the material. Couplants ensure effective contact between the transducers and the material surface to minimize signal attenuation. Couplants can comprise water, oil, rubber, etc. In non-contact-based scanning, the transducers do not directly contact the material surface [9]. Because there is a large difference between the acoustic impedance of air and solid material, non-contact-based UST is more difficult to perform. For example, if the material surface is rough, this approach can be sensitive, and significant energy may be reflected. However, an advantage of this approach is that it prevents surface-near damage. Ideally, the transducers are applied at the ends of the tested member to measure wave propagation along fibers.

**Figure 8:**
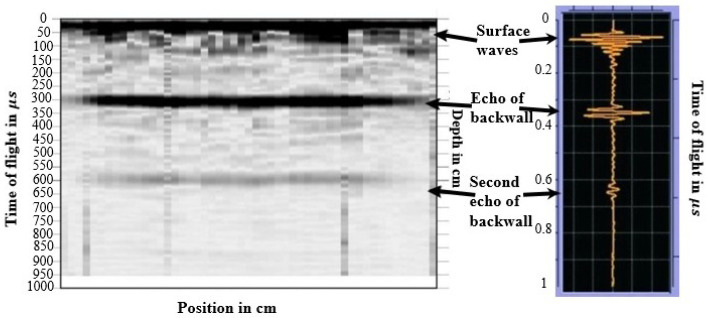
Sample results from UST of a pine specimen: (**Left**) B-scan along the length of the specimen. (**Right**) A-scan at the mid-beam location. Source: [4].

Aicher and Dill-langer utilized UST for glue line defect detection in glulam beams. Depending on the glue line thickness, defect type, and size, the possibilities and limits of transmission and reflection measurements based on compression and shear waves were systematically investigated. The results showed that UST could quantitatively identify the glue line defect for the most realistic glue line defect types [64]. Concur et al. tested UST to detect adhesion issues in CLT panels with varied characteristics. Panel configuration minimally impacted stress wave velocity, but intrinsic wood defects like knots had an influence. This could potentially lead to misinterpretation when assessing gluing defects. Ultrasonic measurements accurately pinpointed unglued regions, offering precise defect detection via velocity maps [65]. Mousavi and Gandomi used UST to detect and classify wood hole damage. Two types of hard and soft wood were used. An advanced signal decomposition algorithm called Variational Mode Decomposition was exploited to derive some features from the recorded ultrasonic signals. Based on the outcomes, the most accuracy was achieved for identifying defects using this method [66]. Zhang et al. introduced a non-contact transverse vibration and UST approach for CLT integrity assessment. These methods are effective for quality assurance post-manufacturing and field diagnostics. UST excels in identifying layer decay and offers detailed layer-specific property data, complementing the transverse vibration method’s overall effectiveness [67]. Mousavi et al. demonstrated the effectiveness of UST and machine learning in standing tree health assessment in the field and lab. The collected signals with UST were processed by variational mode decomposition. The outcomes showed that the proposed method for classifying wood materials based on their health state was effective. This method provided detailed results in the lab and was accurate for in-field specimens; hence, it is considered a reliable method for use on standing trees [68]. Figure 8 shows sample UST results for a Pine laboratory specimen.

Ultrasonic echo results are commonly visualized through A-scans or B-scans. An A-scan (refer to Figure 8 on the right) depicts the transit time and pulse intensity. On the other hand, a B-scan (depicted in Figure 8 on the left) is a composite representation comprising multiple A-scans. This two-dimensional cross-section provides insight into the transit time along the measurement line. A- and B-scans are described in detail in Section 5.1.1. The result reflects the sound wave’s path by multiplying the measured transit time with the known velocity of the sound wave in a specific material. Consequently, a B-scan can directly reveal the inhomogeneities’ location within the specimen. In some cases, the absence of an echo from the backwall may indicate damage within the specimen.

UST offers enhanced penetration capabilities into the wood, allowing for the detection of defects located deeper within the material. Its measurements can provide quantitative information like defect size and location, which can help assess the severity of the defect and inform repair decisions. While less sensitive than the ER method, MC can somewhat impact UST efficiency. Wood’s anisotropy affects the velocity of sound wave propagation, which should be considered in the reconstruction process. UST provides high-resolution data about the internal structure of wood, facilitating accurate identification and characterization of defects. In some cases, differentiating between certain defects based on ultrasonic testing (UST) may be difficult because defects with similar shapes and sizes can exhibit the same signal variation. Ultrasound-based imaging is discussed in detail in Section 5.3.

**Figure 9:**
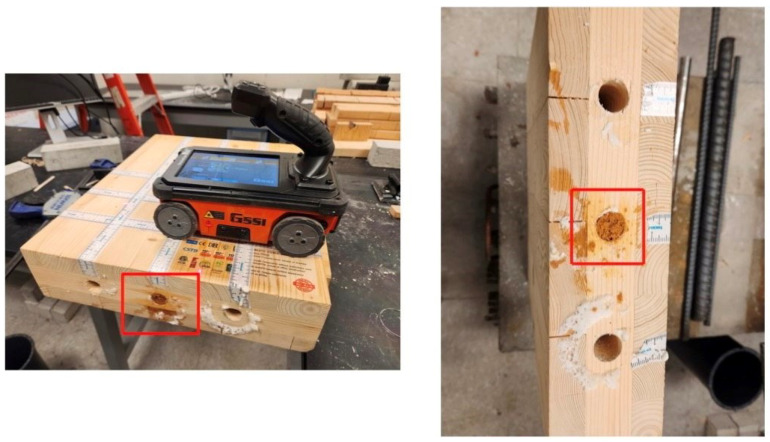
Photos showing authors’ CLT test specimen (**right**) with three holes and GPR instrument (**left**). The middle hole (highlighted with a box) was filled with wet sawdust.

**Ground Penetrating Radar (GPR):** GPR relies on electromagnetic waves (EM) like radio waves or microwaves. Since the inspection depths used in Civil Engineering structures are shallow compared to other uses of radar, these application devices emit short pulses of electromagnetic waves. This is why radar is usually called short-pulse radar or ground penetrating radar [9]. GPR uses electromagnetic waves to investigate the subsurface properties of materials with frequencies ranging from hundreds of MHz to several GHz. Instruments typically consist of three components: a transmitting and receiving antenna (s), an EM pulse generator (within a specific broad frequency band), and a data acquisition unit [24]. Figure 9 shows a 2.7 GHz GPR instrument, and CLT sample with three holes drilled, in which the middle hole is filled with sawdust and the others are empty. The instrument and setup are the authors’ own.

**Figure 10:**
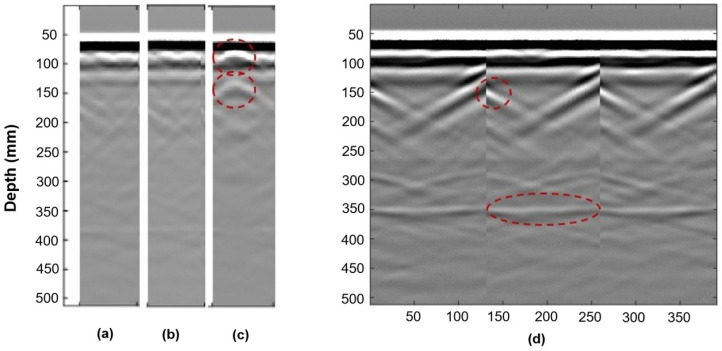
GPR b-scans for (**a**) empty hole, (**b**) hole filled with spray foam, (**c**) hole filled with wet sawdust. (**d**) highlights the reflections from the specimen sidewalls (circle) and a reflection from the laboratory floor (oval). (Results from authors’ work).

Colla used GPR to assess structural timber elements. This method showed detailed information on anomalies and their positions from a laboratory investigation. High-frequency GPR seemed promising for assessing historical timber elements on-site [69]. Brashaw utilized GPR to inspect the longitudinal decks of the timber bridge. Based on the results he provided, GPR had the potential to identify internal defects in timber bridge decks before and after a bituminous layer was added. Void defects that were hollow and filled with foam or sawdust were precisely identified; however, the presence of steel construction screws affected the GPR signal. The ability of this method to identify small and large defects in timber bridges was demonstrated [70]. Wu et al. assessed the internal conditions of timber bridge structural members using GPR. The outcomes showed that GPR contributed to the assessment of the wood structures; it identified the defects’ location and size and differentiated between some categories of defects. Also, the data acquisition was rapid, so this method was the best choice for timber bridge assessment [37]. Figure 10 shows sample results of using GPR on the CLT specimens shown in Figure 9 (authors’ measurements).

The hole filled with wet sawdust has a higher electrical conductivity and, thus, higher relative permittivity than the empty or foam-filled hole. It thus reflects the GPR waves more strongly than the other two. The parabola shapes observable in Figure 10c are caused by the wet sawdust’s reflection of the GPR waves. The empty hole and the hole filled with spray do not result in a detectable reflection, likely because they have (1) similar relative permittivity to wood and (2) are too small. When the radar energy hits an embedded object or void in the specimen, part of the wave is reflected to the antenna, and part continues to travel through the object.

GPR is considered an accurate method for assessing wood structures. It can quantify the location and severity of damage, but the effectiveness of GPR is contingent upon the frequency content of the waves used. However, some factors can affect the quality of GPR images. Denser wood types pose challenges to GPR, resulting in lower signal penetration and potentially less accurate results. In some scenarios, smaller and deeper defects can be more challenging to detect and accurately characterize with GPR. Additionally, differentiating between various defects based on GPR data can be difficult. Finally, GPR is a rapid method (compared to UST) but requires a relatively flat surface to perform a scan.

## 4. Semi-Destructive Testing

**Drilling Resistance (DR):** DR is a semi-destructive technique where a small hand-crank drill or an electrical power drill is used to drive a drill bit into the wood at a constant speed. Although power drilling is faster, hand drilling allows the inspector to monitor drilling resistance and can be more helpful in identifying deterioration pockets. This method has been used in tree growth, bridge, and building investigations. If the drilling needle confronts a cavity or degraded areas, the power input declines, which is visible on the DR profile. Since this is a local test, the findings should not be generalized for larger areas. Also, the needle is usually thin; however, the drilling hole or multiple drillings may be unacceptable for historic timber elements. The outcomes may be affected by factors such as moisture content, drill-bit sharpness, and direction relative to grain orientation [9,39,40,71].

**Figure 11:**
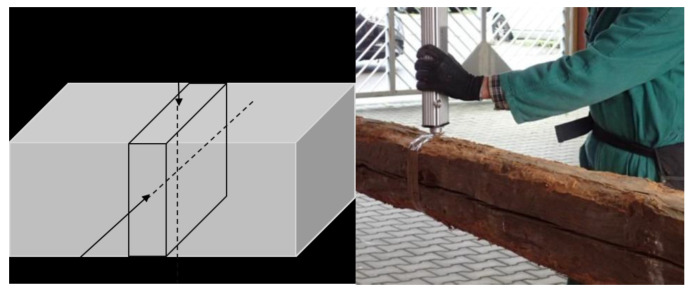
Orientation of the two perpendicular drillings performed at every cross-section. Source: [75].

Nowak et al. evaluated the usefulness of the DR method for the in situ assessment of structural timber. DR was correlated to material hardness density. The results showed that the DR method could estimate the depth of wood decay. There was a need for many measurements to identify the internal defects, and the method should be considered as a qualitative evaluation rather than a quantitative one [72]. Sharapov et al. utilized DR measurements to assess the internal condition of untreated and preservative-treated wooden poles. Based on the outcomes of the DR measurements, they found parts of the untreated wood severely degraded by insects and wood-destroying fungi. In contrast, the treated timber generally showed no reduction in DR. Still, the impact of preservative treatments was not investigated [73]. Frontini used a DR device to evaluate a timber structure in-situ. They proved that this method was practical in evaluating the condition of timber structures. It was shown that some types of degradation, consisting of internal and superficial decay, fungal growth, and cracking, occurred in wood samples [74]. Figure 11 illustrates the directions of the two orthogonal drillings at each cross-section.

**Figure 12:**
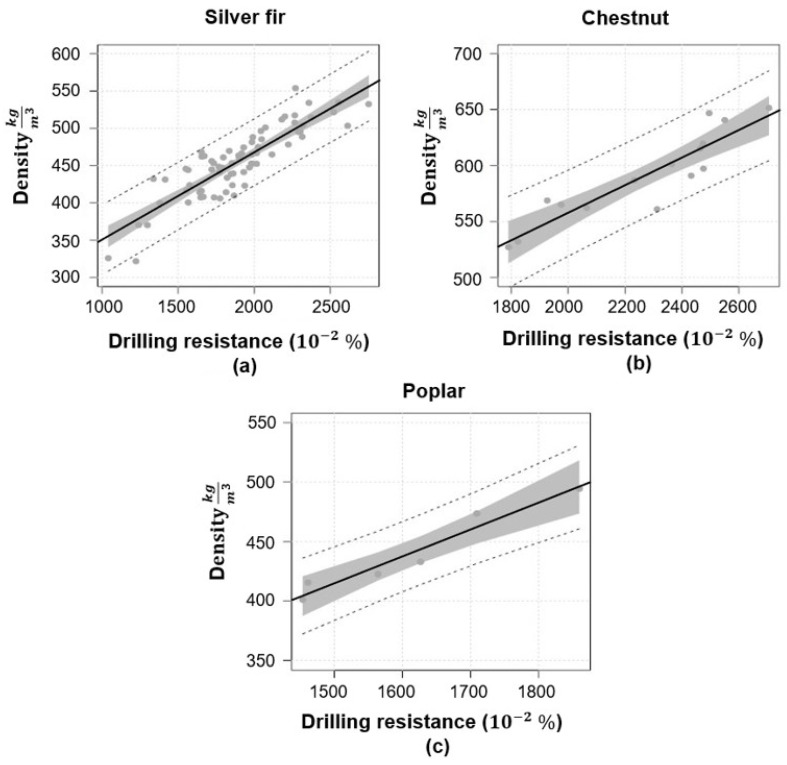
Comparison of different types of wood density and drilling resistance: (**a**) Silver fir, (**b**) Chestnut, (**c**) Poplar. The solid black line depicts the best-fit regression line; the gray shaded area illustrates the 95% confidence intervals; while the dashed lines represent the 95% prediction intervals. Source: [75].

A linear regression analysis between wood density and drilling resistance is shown in Figure 12. The analysis was conducted separately for the three species of Silver fir, Chestnut, and Poplar.

As observed, increasing the density increases the drilling resistance. The average density of chestnuts is higher than Fir and Poplar. However, this technique remains limited, providing only local and one-dimensional information and altering the wood condition. This method can be qualitatively helpful in detecting the presence of defects. However, DR offers limited capability for accurately quantifying the size and depth of defects in wood. Table 3 summarizes all the non-destructive and semi-destructive methods discussed above.

Many techniques that can be applied to wood members in an industrial or research setting are unsuitable for field use. For example, although it provides detailed images for wood inspection, X-ray imaging is challenging due to the need for large, immobile scanners and radiation safety considerations. IRT relies on temperature gradients, which can be affected by external conditions and surface coatings, limiting its effectiveness for wood assessment in outdoor or field environments. Sounding is a straightforward and portable method that can be easily applied. It does not require specialized equipment, making it suitable for inexpensive and rapid on-site wood assessment. However, interpreting sound can be subjective, requiring experience and expertise to distinguish between different types of sounds and their significance. DR can be performed in the field using portable drilling equipment, providing direct access to the internal structure of the wood; however, drilling can be time-consuming and may cause minor damage to the wood surface. The IRT method may not be ideal for outdoor or field assessments because it depends on the presence of temperature gradients, which can be influenced by external factors and surface coatings, potentially limiting its effectiveness in such environments. The UST and GPR methods are robust and can be deployed in the field, providing data while the measurements are taken. Hence, they are selected, and their use for imaging is discussed in more detail.

## 5. State-of-the-Art in Imaging of Mass Timber

Advanced imaging techniques are rapidly transforming how we assess the health of structural timber. Unlike traditional visual inspections, these non-destructive methods, such as UST and GPR, reveal hidden defects like knots, cracks, and decay. These techniques provide detailed visualizations, including defect size and location, allowing engineers to identify potential problems that could compromise structural integrity. This newfound ability to see inside the wood is vital in ensuring timber structures’ safe and efficient design, construction, and maintenance. Additionally, the availability of various imaging techniques, like travel-time tomography and synthetic aperture focusing (SAFT), empowers engineers to choose the most suitable method for a specific assessment [2,82].

### 5.1. Fundamentals of Imaging

This section lays the groundwork for understanding how various methods provide insights into the internal structure of timber members. Hence, the visualization of data collected during imaging using A-, B-, and C-scans will be explored, revealing different perspectives on the material’s structure. Advanced processing techniques, such as SAFT, which refine these data for sharper images, will then be investigated. Additionally, tomography methods employing sound or microwaves will be examined, offering a direct view of the material’s internal composition. Finally, Full Waveform Inversion (FWI), a technique used in geophysics that shows promise for GPR-based defect detection in trees, will be discussed. In this section, you will be provided with knowledge of these imaging tools, facilitating a deeper understanding of their contribution to timber assessment.

#### 5.1.1. A-, B-, and C-Scans

**Figure 13:**
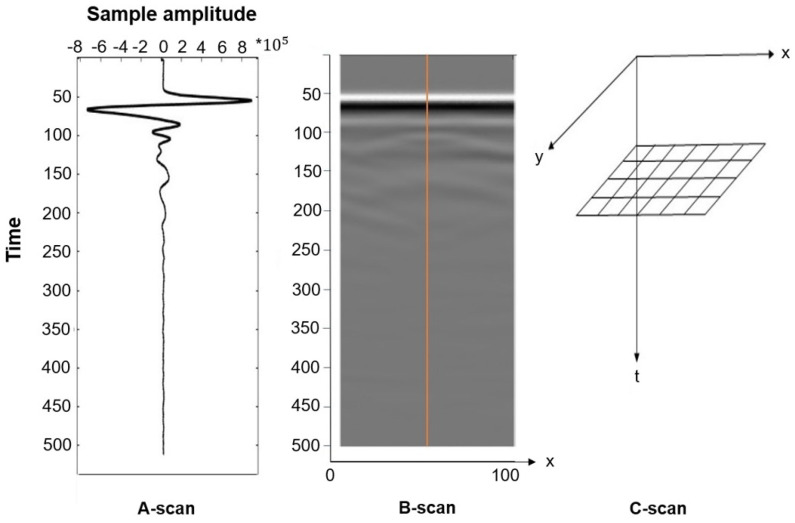
Basic data display modes illustrated with sample GPR measurements: A-scan, B-scan, and C-scan.

Basic data display modes include A-scan, B-scan, and C-scan. The A-scan (amplitude mode display) shows signal amplitude vs. sampling time. It represents a single measurement and can be used to determine the time of flight to assess the location of a discontinuity within the material. The signal amplitude can also be used to estimate the discontinuity’s size. This is achieved by comparing the signal’s amplitude reflected from an unknown reflector (such as a defect) to that from a known (=reference) reflector [83]. The B-scan (brightness mode scan) is a composition of various A-scans recorded at defined distances. The B-scan represents a 2D cross-section through the specimen and enables the identification of a change in signal structure along the measured axis. It helps to determine the depth of the reflector and its approximate linear dimensions in the scan direction. If several B-scans recorded with a defined offset are combined, it is possible to interpolate a horizontal layer. An interpolated layer (C-scan or motion mode) can give 3D information about the structure and damages in the object [18,84]. Figure 13 shows all three display modes using the authors’ GPR measurements of the CLT sample shown in Figure 9.

#### 5.1.2. SAFT

**Figure 14:**
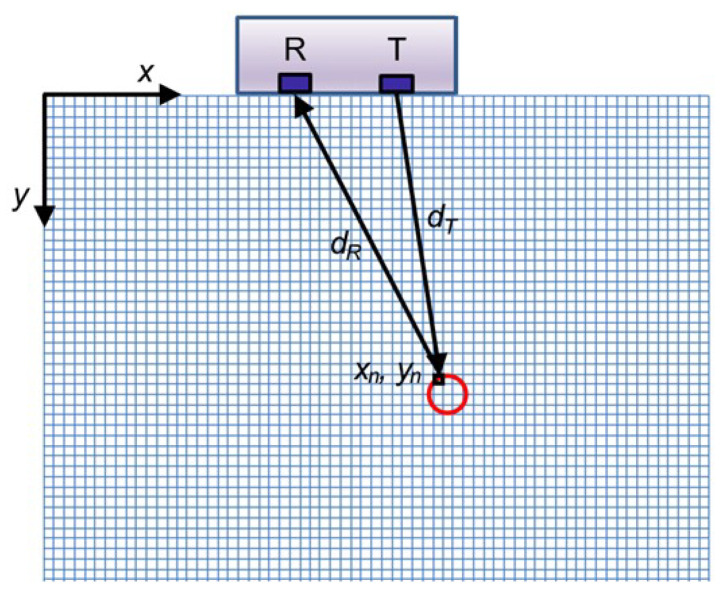
Visualization of the SAFT process for a specific instrument location and pixel. The grid outlines individual pixels, with ‘T’ representing the transmitter and ‘R’ the receiver. For simplicity, only two transducers are depicted. Source: [89].

A-scan data are recorded using GPR and UST to scan a timber member. While B-scans allow evaluating a cross-section, more advanced algorithms can further process the collected data to produce a detailed image that shows the reflectors in a tested member more precisely. The most commonly used imaging algorithm is the synthetic aperture focusing technique (SAFT), which maps the amplitudes of the A-scans onto a finite grid of a depth slice, assuming that every point on the grid is a potential reflector. This process is by some referred to as “migration”. By using arrays of transducers, the time of data collection is reduced while data density is increased, which results in images with a better resolution. SAFT utilizes pulse-echo data for all elements of the array transducer [85]. Each element emits and receives signals in this process, which is repeated for all elements. Array data also allows the use of the total focusing method (TFM), an advanced imaging algorithm that leverages all the information within the full matrix of array data. TFM is called multi-static acquisition, resulting in significantly more data than the mono-static case (which employs one emitter and one receiver per measurement location). The reconstruction is then applied to the collected data, following the same mono-static and multi-static cases principles. SAFT and TFM rely on coherent amplitude summations to generate detailed output images. This procedure is equivalent to focusing at each point (pixel) of the reconstructed image by computing the proper delays [86]. Delays are calculated by determining the distance from the emitting to the receiving transducer and dividing it by the wave velocity [86,87,88]. The intensity of the image, IM, over a region of interest ($x_{n}$, $y_{n}$) is reconstructed using the following equation:

(4) $$\begin{array}{cc} IM(x_{n},y_{n}) & =\sum_{i=1}^{Nch}\sum_{j=1}^{Nch}X\left(\left(\frac{d_{T}+d_{R}}{V}+\epsilon \right)\times F_{s}\right),\;\\ d_{T} & =\sqrt{{\left(x_{n}-x_{T}\right)}^{2}+{\left(y_{n}-y_{T}\right)}^{2}},\;\\ d_{R} & =\sqrt{{\left(x_{n}-x_{R}\right)}^{2}+{\left(y_{n}-y_{R}\right)}^{2}} \end{array}$$

where “*X*” is an individual measurement (or A-scan), $d_{T}$ and $d_{R}$ are the distance from the transmitting transducer (at $x_{T}$, $y_{T}$ = 0) to the pixel considered (at $x_{n}$, $y_{n}$) and the pixel of interest considered to the receiving transducer (at $x_{R}$, $y_{R}$ = 0), respectively. “*Nch*” is the number of channels (or transducers), “ϵ” is a time offset, “*V*” is the wave speed, and “$F_{s}$” is the sampling frequency. Both “ϵ” and “*V*” need to be determined experimentally. Figure 14 illustrates this process for a select location of the instrument and the pixel considered. Only two transducers are shown for simplicity; however, this technique can be used for arrays with any number of elements.

In SAFT, the depth slice along the scan profile is divided into small pixels on a regular grid. When the amplitudes observed at a given grid position measured from different locations coincide with an actual reflector, they result in constructive interference, and the pixel is illuminated, highlighting the reflector. When all array elements participate in emitting and receiving signals, so-called Full Matrix Capture (FMC) can be achieved, whereby collected (unprocessed) data are reshaped into full matrix form with the diagonals being zeros and the matrix being symmetric [86,88]. The diagonals are zeros when transducers cannot transmit and receive data simultaneously. Half-matrix Capture (HMC) is achieved when only one signal is recorded between each transducer pair. This is common for ultrasonic arrays used for concrete applications [90]. Each measurement location produces n(n−1)/2 A-scans for this case. A vectorized version of the TFM, which is computationally efficient, can be implemented [91].

Previous studies indicate that the choice of imaging algorithm influences the quality of the reconstructed images. While TFM provides ample resolution, its computational complexity results in significantly longer processing times, especially when applied in real-time applications [85]. SAFT is chosen for reconstructing images due to its flexibility in transmitter and receiver combinations during scanning and relatively efficient calculations. However, there is room for enhancing the image quality of SAFT. Given its shorter processing time, its computational efficiency is a strength, as it involves calculating only one transmitter-receiver pair for each element, resulting in fewer computations than other algorithms [92]. One limitation of traditional SAFT is its reliance on a single wave velocity, which can cause problems for anisotropic media such as wood or FRP materials. Another factor limiting the resolution of traditional SAFT is its use of a single transducer, particularly compared to approaches that use ultrasound-phased array probes, which are common in the medical field and for testing metals [93]. A drawback of TFM is its extended duration, attributed to its extensive data collection process [94].

#### 5.1.3. Tomography

**Figure 15:**
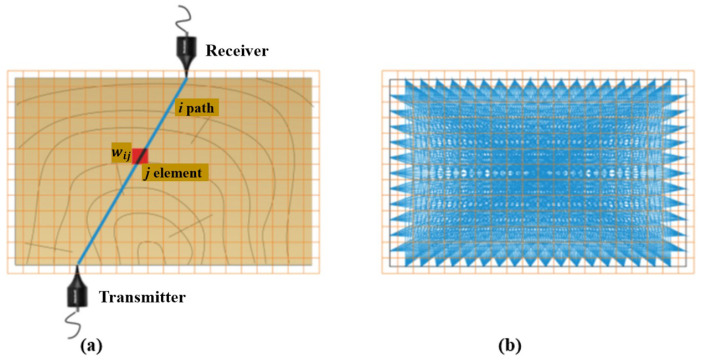
Tomography scheme illustrating direct wave ray paths: (**a**) a path through element ‘i’ passing ‘j’, and (**b**) multiple paths through the object. Source: [28].

Tomography utilizes penetrating waves such as sound waves or microwaves to create images of the internal wooden elements. Different types of tomography techniques exist, including ultrasound tomography, considered the most common method. While SAFT images show the reflections from boundaries between materials with dissimilar properties, tomographic images represent a velocity distribution map, representing the actual inclusions. They, thus, are more direct representations of the cross-section. In travel time tomography, the desired section is divided into finite elements to produce tomographic images, with transducers placed at several locations, as shown in Figure 15a. The final resolution of images depends on the indicated elements. The size of the elements depends on the geometry and density of the ray path pattern (see Figure 15b).

$w_{ij}$ is the length of the *i*-th direct wave travel path passing through the *j*-th pixel. As shown in Figure 15, this division results in several ray paths corresponding to specific measurements, from the emitter to the receiver transducers; each ray travels through specific elements at varying distances. The total recorded travel time, or time-of-flight (TOF), represents the average velocity from the emitter to the receiver transducers [95,96]. The system of linear equations used to reconstruct the velocity profile can be expressed as

(5) $$\begin{array}{cc} t_{i} & =\sum_{j=1}^{n}w_{ij}s_{j},\;i=1,2,3,\ldots ,m,\;j=1,2,3,\ldots ,n\; \end{array}$$

where $t_{i}$ represents the total time from the transmitter to the receiver for the *i*-th path passing through the *j*-th pixel, and $s_{j}$ denotes the ultrasonic pulse slowness, which is associated with the ultrasonic pulse velocity $v_{j}$ in pixel *j*, as follows:

(6) $$\begin{array}{cc} s_{i} & =\frac{1}{v_{j}}\; \end{array}$$

The velocity in each pixel is determined based on the cross-sectional geometry [28]. In one study, the highest velocity is observed when waves propagate through the pith [28]. Evaluating the attenuation levels in specific material regions compared to a reference (undamaged) area allows for a relative damage measurement. Also, by comparing the TOF values of specific points in the material with a reference value, it becomes possible to indirectly estimate the severity of damage through the analysis of deviations in travel times. Therefore, it can be concluded that internal damage within the wood or material significantly impacts wave propagation, resulting in decreased velocity and increased attenuation in the damaged region [97]. In recent years, it has been shown that tomographic imaging works on larger components and can be easily utilized to image in-service structural timbers [82]. The velocity data from tomography can identify timber areas with different properties, such as knots, cracks, decay, or insect damage. Ultrasonic tomography is a diffraction-type tomography that is noninvasive and safe at low energy levels. For the tomographic analysis and reconstruction of images, reflection data collected by illuminating the sample with multiple scans from different directions around the specimen are gathered. All characteristic parameters, such as TOF, amplitude, frequency, etc., can be used in data collection [9]. In ultrasonic tomography, the detection resolution highly depends on the wavelength of the generated waves and the density of the ray paths. Short wavelength comes from high frequencies of ultrasonic waves; thus, they are more sensitive to subtle defects and provide a highly detailed image. On the other hand, high frequencies attenuate more quickly, which limits the size of the element studied [44].

Perlin et al. presented an advanced method for ultrasonic tomography investigations. This method considered the wood anisotropy and the orthotropic behavior along the tangential and radial directions with the growth ring direction. The Christoffel or Hankinson equation was utilized to model the orthotropic behavior of wood. The tomograms demonstrated the capability of this method to identify the internal flaws in wooden elements when the anisotropy was considered [98]. Zielinska and Rucka studied wooden beams retrieved from a historic building. The effect of wood condition on the ultrasound TOF was assessed, and the images from tomography were compared with visual inspection results. Based on the results, the places of discontinuities, voids, and cracks resulted in a lower wave propagation velocity, and the wood core location showed the highest velocity [99]. Espinosa et al. aimed to identify the presence of pests and diseases in standing pine trees using travel-time computed tomography (ultrasound-based method). An iterative method was developed to consider the curved ray paths caused by the wood’s anisotropic. The obtained velocity images showed that the proposed method can locate the defects more accurately than standard approaches. Hence, the anisotropy of wood should be considered in image reconstruction algorithms for ultrasound computed tomography [97]. Zielinska and Rucka utilized ultrasonic transmission tomography for the in situ assessment of wooden beams of historical buildings for imaging of the internal elements. In this research, the travel-time ultrasonic tomography technique was used to achieve the internal image of a wooden element in terms of the velocity of an elastic wave propagating within the medium. The outcome image showed that ultrasonic tomography effectively indicated the damaged locations in the beams. However, the anisotropy of wood resulted in the bending of wave paths [28].

#### 5.1.4. Full Waveform Inversion Imaging

Full-waveform inversion (FWI) is a technique mostly used in geophysics. FWI iteratively estimates the subsurface properties of a structure by minimizing the misfit between observed and modeled waveforms. It utilizes the entire waveform recorded by seismic or other wave-based sensors, capturing detailed information about the interaction between waves and subsurface structures. The idea of FWI is to adapt the parameters of an initial simulation model of the undamaged specimen by minimizing the discrepancy between these simulated signals and experimentally measured signals of the flawed specimen [100]. While having found some applications for concrete members [101,102,103], the use of FWI to study timber structures is not common owing to the complex nature of wood, which makes it difficult to model the ultrasonic wave propagation accurately and to obtain reliable inversion results. However, in a recent article [104], a GPR multifrequency FWI algorithm is considered both permittivity and conductivity to aid in tree protection and restoration, particularly in detecting trunk defects. The method utilized total-variation (TV) regularization and surface-coupled commercial GPR common-offset data. Numerical and field experiments demonstrated the potential of this approach in detecting trunk defects. The study accurately reconstructed the internal structure and defect details, reducing uncertainty and ambiguity in defect detection.

### 5.2. GPR-Based Imaging

GPR-based imaging uses radar technology to create images or maps of objects or scenes. It utilizes electromagnetic waves to detect and locate anomalies, moisture content, and fiber direction in materials like wood. Basic GPR instruments have one transmitting and one receiving antenna housed in one instrument at a fixed distance. The transmitter emits the waves into the material, and the wave energy is scattered or reflected toward the receiver. Using SAFT (see Section 5.1.1 and Section 5.1.2), the recorded waveforms are converted into images [24,37,105]. Damage identification is done by comparing the signal’s amplitude reflected from an unknown reflector (such as a defect) to that from a known reflector. The difference in signal amplitude can estimate the discontinuity’s size. While three-dimensional representations are also possible, they are not discussed here since they are not commonly used for wood applications. A photo of a 2.7 GHz instrument is shown in Figure 9 (left).

**Figure 16:**
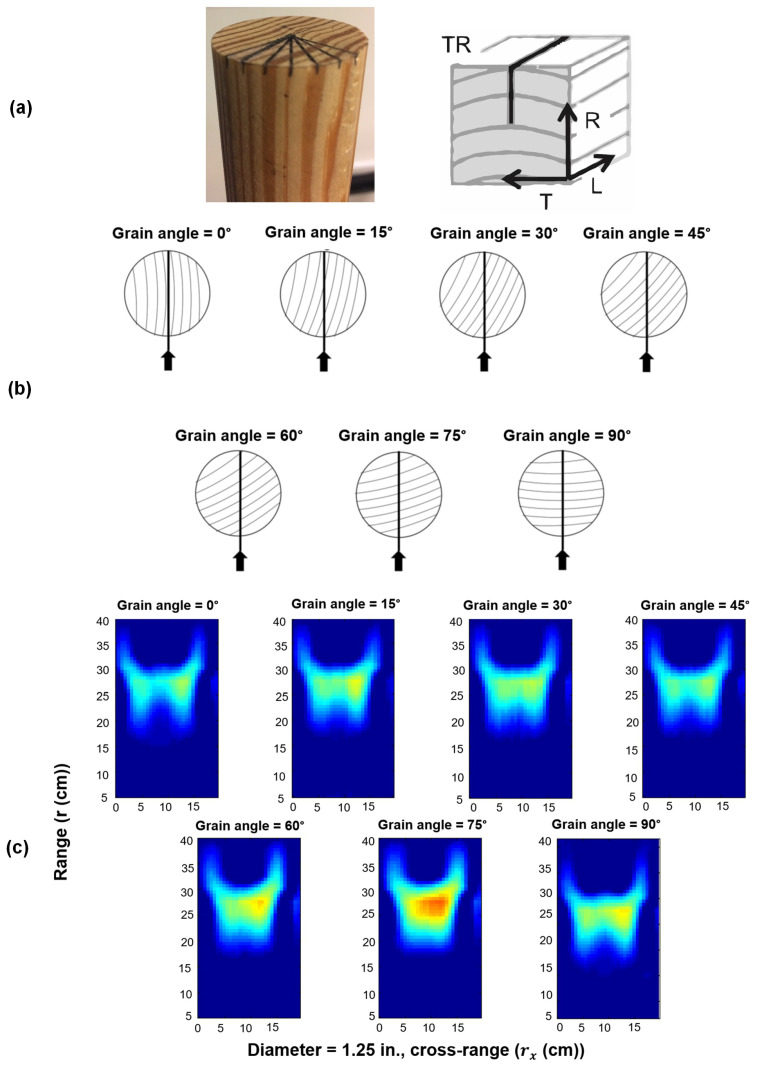
(**a**) Grain angle distribution in the TR plane, (**b**) grain angle orientation relative to the imaging radar, ranging from 0° to 90° in the positive direction, (**c**) SAR images for d = 1.25 at GA = 0°, GA = 15°, GA = 30°, GA = 45°, GA = 60°, GA = 75°, and GA = 90°. Source: [106].

Ingemi and Yu used the Synthetic Aperture Radar (SAR) imaging technique to evaluate changing grain angle in cylindrical pine elements. Two wooden samples were employed, each measuring 14 inches long with diameters of 1.25 inches and 1.5 inches. Positioned vertically within an anechoic chamber, each sample underwent imaging at various orientations via a 10 GHz SAR system. Optimal outcomes were achieved by maintaining a distance of 25 cm between the specimens and the instrument. SAR was considered an effective method for subsurface evaluation because it can be performed remotely and has high resolution. On the other hand, electromagnetic methods entail a contact or near contact inspection condition, but by using SAR, there is considerable freedom to perform inspection during the measurements. Using the output images, they found that the grain orientation of the wooden elements affected both the integrated amplitude and amplitude distribution. Hence, it is confirmed that SAR imaging could be useful for detecting the grain angle in structural timber. Sample images from this research are presented in Figure 16 [106]. Hernandez and Duwadi used Micropower Impulse Radar (MIR) technology to image structural timber. Their imaging system is portable, lightweight, battery-operated, and a relatively inexpensive tool. The radar data were collected by scanning the radar unit over the wood surface and performing measurements at uniform intervals. The multi-frequency diffraction tomography algorithm was utilized to map the acquired data into high-resolution images. The results showed that the MIR imaging capability was high enough to illustrate the extent and location of inhomogeneities in wood and produce data that could be easily interpreted. However, there was a need to optimize the image-reconstruction algorithm or a post-processing algorithm to filter out unwanted noise [107].

**Figure 17:**
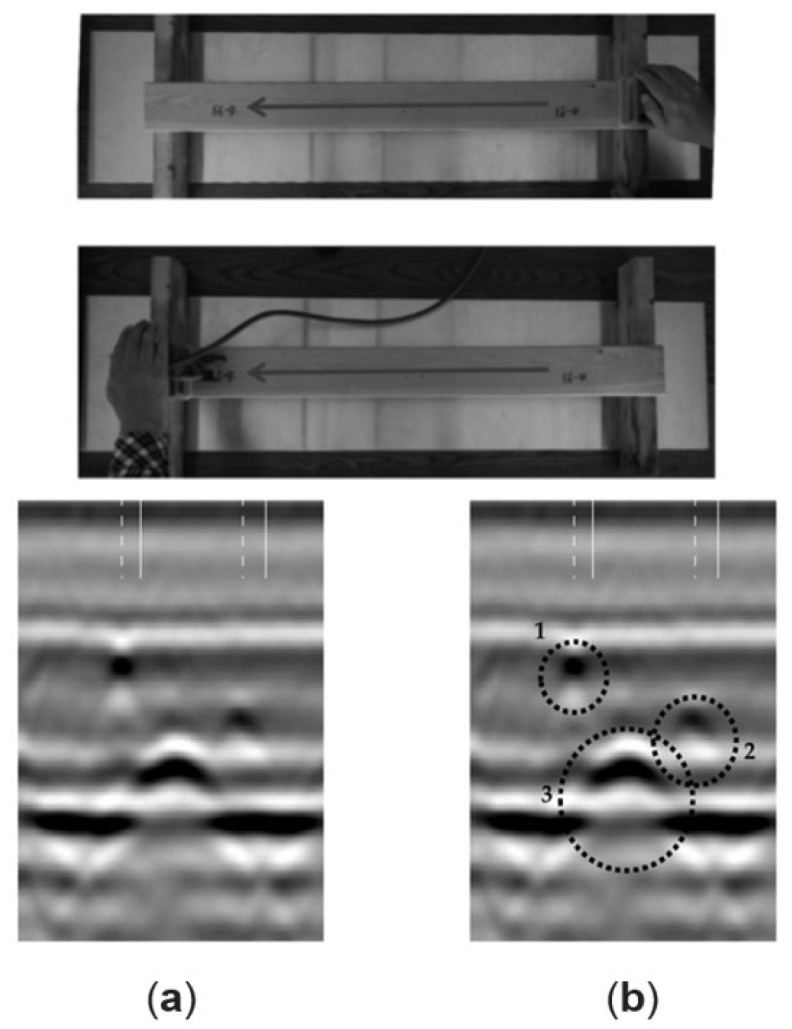
(**a**) Radargram (B-scan) illustrating a typical Ash specimen. In (**b**), three internal features are outlined by dotted circles. Features 1 and 2 indicate internal knots, while Feature 3 denotes a circular void intentionally drilled into the wood. Source: [24].

Figure 17 shows a sample GPR B-scan of a 3.8 × 8.9 cm Ash specimen positioned directly over an aluminum sheet. Although all three features were clearly visible and easily located, determining their nature was challenging.

**Figure 18:**
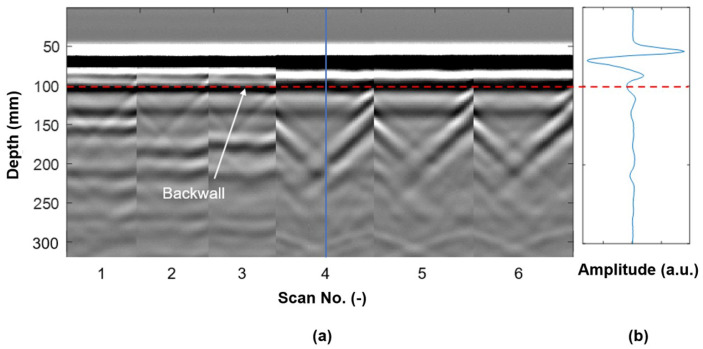
(**a**) GPR B-scan recorded on Spruce CLT specimen by the authors includes three measurements in each the longitudinal and transverse direction on the specimen surface, (**b**) sample A-scan corresponding to the blue line on Scan 4.

Figure 18 shows a series of GPR B-scans recorded on a Spruce CLT specimen with a thickness of 100 mm, consisting of five cross-laminated layers without any holes. The length and width of the specimen are 400 mm and 500 mm, respectively. The backwall reflection is visible as a dark line, indicating a reflection from an air boundary. Note the additional reflections and artifacts that require experience in assessing B-scans.

Over the past two decades, GPR scanning has been utilized for identifying defects, anomaly localization, and characterization of various materials such as sand, concrete, and wood [108]. The production of GPR waves at high frequency leads to a short wavelength; whenever the wavelength is larger than the dimension of the heterogeneity, the resolution can be low, and sometimes damage identification may not be possible [9,69].

However, several characteristics make the GPR a noteworthy NDT tool for timber inspection [24]. One of the major attributes of GPR is its penetration into the subsurface and identification of unseen conditions. Additionally, it can quickly scan large surface areas compared to some acoustic or stress wave techniques, necessitating point-by-point inspection. This ability is important for some structures like timber bridges, where time out of service can be immensely disruptive. GPR is sensitive to subsurface moisture and embedded metals [109]. For structural application, the GPR instruments are portable, can be applied using one hand, and there is no need for special safety arrangements due to harmless waves compared to other methods like X-ray [39]. Also, it can help estimate feature depth in addition to location. Data collected can be displayed in 1D, 2D, and 3D images showing the inspected object’s internal structure, as seen in Figure 13. The main advantages of GPR are its cost-effectiveness for initial surveys and its time efficiency due to rapid data collection. It is particularly well-suited for extensive coverage of large areas.

While locating internal features using GPR images may seem intuitive, identifying the nature of a detected defect (such as a knot, void, or metal connection) and distinguishing these features have proven difficult because they have a similar appearance in GPR radargrams [24]. Despite this limitation, GPR does offer the ability to detect moisture pockets, often serving as an indirect indicator of internal decay, as discussed in Figure 10. Current research in locating decayed wood using GPR is limited, with inspections relying on moisture presence as an indicator, even though decay may persist in the absence of moisture. A crucial advancement would involve identifying and locating internal decay based on distinct characteristics in the GPR signal, independent of moisture presence [108]. As stated in Section 2.2, the propagation of radar waves through wood is influenced by several factors such as wood density, variation of moisture in wood, temperature, specimen size, and shape, size of the internal damage, radar wave frequency, preservative treatments, and a combination of these factors [109]. Whenever the permittivity increases, the absorption and conductivity also increase. Therefore, GPR waves are quickly attenuated, and the penetration depth declines. The applied frequency is critical because it affects the penetration depth and resolution of the image. GPR waves with higher frequencies offer better resolution. However, the downside of higher frequencies is that they are more susceptible to attenuation in wood, especially moist wood. As frequency decreases, penetration depth increases, but the detectable minimum size defect increases. There are several user-configurable settings for GPR, like gain and frequency pass filters, which can affect the inspector’s ability to locate and detect internal features. To accurately determine the depth of defects, cracks, or connections using a B-scan or reconstructed image, parameters like wave velocity and any time off-sets need to be determined experimentally [24]. The scanning process effectively detects substantial defects characterized by hollowness and adhesive filling. Nevertheless, smaller defects, particularly those with a size similar to the wavelength, may pose challenges in discernibility. The geometric attributes of the defects also play a significant role in the clarity of signal reflection. Some research showed that defects with a round shape may exhibit less distinct identification than those with a straight horizontal boundary between sound wood and a defect void. When metal is present within the wood, optimal scanning results are achieved by aligning the scanning direction perpendicular to the orientation of any embedded metal hardware, such as screws or anchors. This ensures that the electromagnetic waves interact with the metal to facilitate accurate detection. If the wood surface is covered with a layer of bituminous or other materials, the signal response is dampened, making it more difficult to detect a defect [24,70,110].

### 5.3. Ultrasonic-Based Imaging

Many measurements must be performed to produce ultrasonic-based images. Generally, a transducer emits an ultrasonic pulse into the target area or object. The resulting waves propagate through the medium and interact with the internal structure. Their energy is partially reflected when ultrasonic waves encounter boundaries between materials with dissimilar acoustic impedances. Measurement setups range from simple two-transducer pitch-catch setups to multi-channel arrays with multiple transducers per channel. Measurements are typically conducted from one surface, relying on reflections. Receiving transducers detect the waves arriving at the surface and convert them into electrical signals [111]. These ultrasonic waveforms (or A-scans) are processed and used to reconstruct an image using the methods discussed in Section 5.1.2 and Section 5.1.4. To produce tomographic images (see Section 5.1.3), through-transmission is required, which requires that transmitting and receiving transducers are deployed on opposite sides of a tested member.

**Figure 19:**
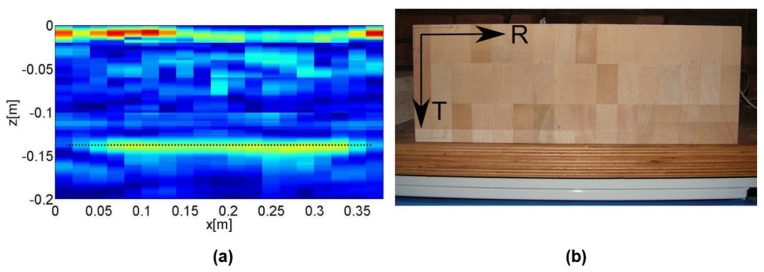
Sample SAFT results: (**a**) anisotropic 2D SAFT reconstruction for Beech (backwall indicated by dotted line), (**b**) RT (radial and tangential) section (figure content courtesy of M. Krause, P. K. Chinta, K. Mayer, U. A. Effner, and S. Müller).

Krause et al. utilized ultrasonic imaging to identify defects in wooden members and glued-laminated timber specimens. They developed a 3-D SAFT imaging scheme for synthetic data from modeling elastic wave propagation. The results showed that low-frequency ultrasonic echo measurements correctly imaged artificial reflectors using interactive reconstruction software called Elastodynamic Finite Integration Technique (EFIT) based on the principle of SAFT. It is mentioned that further research is required to increase the imaging depth of glulam made from spruce, and further investigation is needed to explore the impact of the relationship between wave velocity and direction directly in the SAFT image intensity [112]. Fang et al. investigated whether combining compressed sensing techniques with air-coupled ultrasound (ACU) imaging is possible. They aimed to reduce the number of scanning lines to speed up the imaging process. At first, to overcome the restriction of the compressed sensing framework, they proposed an undersampled scanning strategy specified by a random binary matrix. Implementation was straightforward and required only minor modifications. Then, discrete cosine transforms (DCT) were selected experimentally as the representation basis. Afterward, an orthogonal matching pursuit (OMP) algorithm was used to reconstruct the wood images. They also used the peak signal-to-noise ratio (PSNR) to measure the reconstruction quality. Finally, the experiment was conducted again using the discrete Fourier transform (DFT) and the discrete wavelet transform (DWT), and the results were compared to the DCT. The outcomes illustrated that the same quality of ACU images was obtained by reducing the overall scanning time by half. Moreover, the DCT basis reached the highest PSNR [113]. Vossing et al. performed imaging on wood panels using air-coupled ultrasound (ACU) to identify wood defects. They used cellular polypropylene (PP) transducers with a high signal-to-noise ratio. The PP transducers had a low modulus of elasticity and low density, leading to a small difference in acoustic impedance for transmitting ultrasonic waves between the transducer and air. The specimens were selected from multiplex, LVL, and MDF, and a quasi-peak-detector was used to record data. The results showed that although the signal may be weak because of wave scattering on a sound path in the specimen, the main defects and defect depth could be calculated by measuring the signal’s TOF. The inspection speed was high, and there was less noise interference [114]. Krause et al. presented the development and the first application of ultrasonic imaging of timber using 3D-SAFT. The tests were performed on pine and beech specimens, and the highly anisotropic ultrasonic velocity of wood was considered in the algorithm. The anisotropic SAFT reconstruction was applied to both the measured and synthetic data, which was generated using elastodynamic finite integration technique (EFIT) modeling. Using isotropic SAFT, defects were not identified in the correct position; however, using anisotropic SAFT resulted in the localization of all the inhomogeneities at their correct positions [111]. An example of a specimen glued from small rectangular bars (size about 2×5×50 cm^3^) using melamine resin glue, L-axis perpendicular to the image plane, is shown in Figure 19.

**Figure 20:**
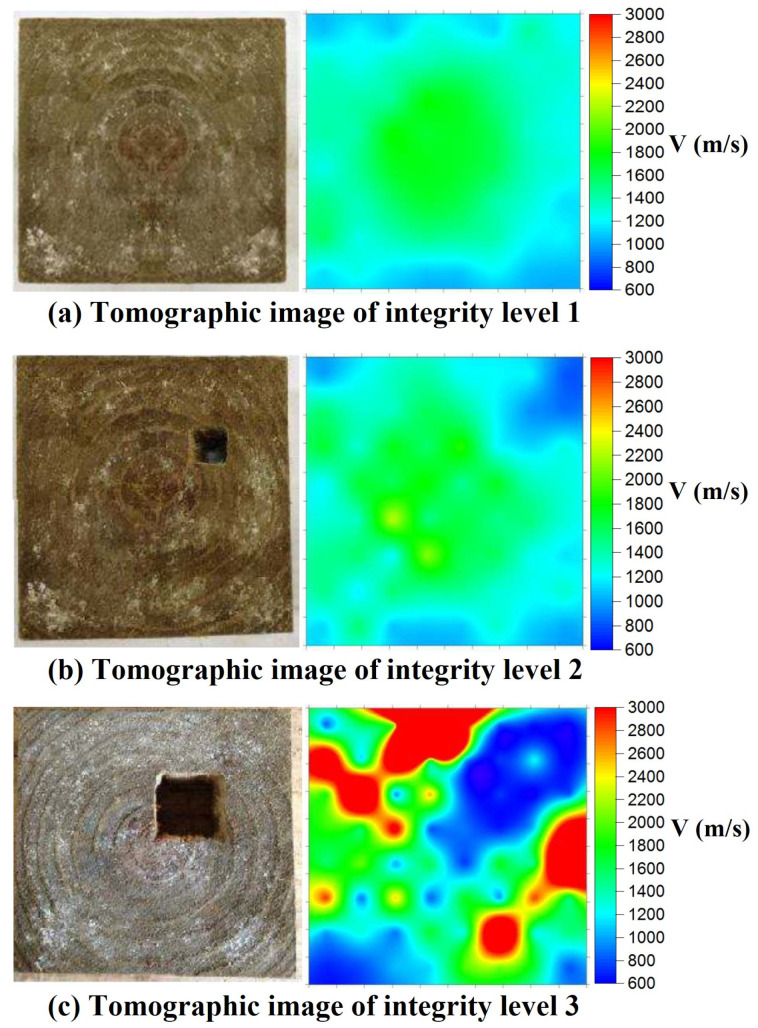
Sample tomographic results presenting both a photo and corresponding tomogram. (**a**) an intact, relatively homogeneous section, (**b**) Level 2 integrity with one element cut out, and (**c**) Level 3, indicating a broader area cut out, suggesting some lack of homogeneity in the region. Source: [96].

Figure 20 shows how a void or discontinuity in a live section of a Pinus tree affects the velocity distribution in a tomogram [96].

Figure 20a illustrates an intact section with relatively uniform characteristics, as expected for integrity at level I. A higher ultrasonic pulse velocity can be observed in the central part of the section. Moving to level II of integrity, where one small element was removed, Figure 20b reveals a small region with lower velocities in the upper right corner near the removed element. Transitioning to level III of integrity, Figure 20c unveils a more extensive area with reduced velocities, indicating a lack of homogeneity in the region. Note the regions with elevated velocities that are not present in previous images. It is worth noting that the tomography algorithm employed is designed for isotropic materials, and the high-velocity areas in certain regions of Figure 20a,c may be linked to the assumed ray paths traveling in a straight line, aligning closely with the radial direction of the fibers in the wood element.

This method provides direct and relatively affordable localization of major defects such as air voids in timber. It allows for higher resolution and thus more precise location of wood defects compared to GPR [115]. Because of the anisotropic nature of wood, measurements usually take longer than in materials with isotropic properties. This is because fully characterizing the ultrasonic behavior of wood requires measurements in multiple directions. Also, ultrasonic wave attenuation is relatively high. Some research states that the anisotropy and heterogeneity of wood lead to inaccuracies and artifacts in the images, such as a shift of defects in the images or loss of focus if not considered properly. However, using SAFT, which can account for anisotropic behavior by considering velocity differences, focus, and accuracy, can be improved [18]. A comparison between UST employing longitudinal (or compression) waves and UST with transverse (or shear) waves showed that longitudinal waves are more sensitive to a crack than transverse waves. This attribute also yields the bending of ultrasonic wave paths [28,99,116]. As discussed in Section 2.3, the transmission of mechanical waves in timber is affected by many factors, such as the frequency range, moisture content, temperature, etc. There is a trade-off between the depth to which the waves may travel (penetration depth) and the level of detail they can reveal (resolution) based on the chosen frequency. Lower frequencies can penetrate deeper but offer less detail. Generally, higher frequencies experience higher attenuation. Moisture acts like a damper for ultrasonic waves, causing attenuation of the signal. Also, any defects will result in additional attenuation because of wave scattering and reflection. This matter makes interpreting results more difficult. Also, some wood features such as knots, grain orientation, and annual rings result in anisotropy and heterogeneity, so the measurement becomes complicated since they further affect the propagation of the ultrasonic waves (wave attenuation) [114,117]. The inadequate coupling of transducers can lead to additional uncertainty in the measurement; therefore, the transducers should have adequate and consistent coupling with the tested member; by doing so, the delay in transmission can also be minimized [9,62]. Ultrasonic-based imaging enables the detection of internal decay, identifying defects, and estimating their size, shape, and characteristics. However, in some instances observed during an experiment, this method tended to overestimate the size of defects in certain wood sections where cracks were present [118]. At a frequency of 100 kHz, damage smaller than 10 mm in diameter could not be identified directly. In addition, in some cases, UST could not differentiate between different types of defects, which can be an important criterion in NDT methods [4].

## 6. Conclusions and Outlook

This article provides an overview of non-destructive testing (NDT) methods applied to wood inspection, specifically focusing on ground penetrating radar (GPR) and ultrasonic testing (UST)-based imaging for application to structural timber. Several aspects make these techniques attractive inspection tools for wood structures.

A wide range of GPR instruments are commercially available and portable, enabling rapid data collection. Due to its sensitivity to moisture, voids, and metal inserts like anchors or connectors, GPR is a valuable technique for examining wood structures. It can potentially detect deep cracks due to its long signal penetration depth, but its resolution might be insufficient for a precise characterization. Lower frequencies offer greater penetration depths at the expense of resolution. GPR is the more cost-effective option for initial surveys or scanning extensive areas than UST. The presence of certain surface coatings can make defect identification with GPR challenging. Dielectric properties govern electromagnetic wave propagation, making it a direct indicator of moisture content variations. While grain orientation and annual rings impact GPR measurements, their resolution is typically insufficient to distinguish these features. As an example, at a central frequency of 1.5 GHz, the penetration depth of GPR in highly moist materials is typically less than 0.40 m.

Although data collection with UST is slower compared to GPR, it is more effective in detecting deep cracks, delaminations, back walls, and inclusions such as air (e.g., voids or insect holes) or regions of low density (e.g., pith or rot), which all offer a strong contrast in acoustic impedance. UST indirectly indicates moisture content variations by changes in the ultrasonic (US) wave velocity; higher moisture content results in increased wave propagation time, with velocities decreasing rapidly up to approximately 30%. Grain orientation and annual rings strongly influence UST, potentially leading to image distortion or artifacts. US waves travel more efficiently along the grain due to a rigid and uniform path, acting as a waveguide. US wave velocities generally decrease with an increased number of rings, but this trend varies among wood species. For example, only Scots pine and Cedar exhibit a gradual decrease in US wave velocities with increased annual rings. A pulse frequency of 125 kHz or lower is recommended to minimize wave scattering and achieve sufficient penetration depth for UST.

Utilizing imaging algorithms can enhance the interpretation of collected data from GPR and UST measurements, reduce artifacts and noise, and produce clearer visual representations of a timber member’s internal structure in the form of 2D or 3D reconstructed images. The synthetic aperture focusing technique (SAFT) and total focusing method (TFM), an extension of SAFT, can augment UST and GPR for wood inspection due to their suitability for rapid data collection and availability. Assuming a single velocity value for imaging can lead to inaccuracies, blurring, or distortion of features. SAFT addresses some of the limitations of UST and GPR measurements by improving resolution, particularly when anisotropy is considered, which leads to a more focused and accurate image. Using low-frequency UST that integrates longitudinal and transverse waves alongside advanced reconstruction software like SAFT and TFM has proven effective in imaging internal features and defects within wood. While SAFT enhances image quality, it does not directly address the fundamental accuracy challenges inherent in GPR and UST measurements for wood. Because SAFT-reconstructed images rely on measurements taken from one surface only, i.e., these are reflection- (or echo-) based measurements, they cannot be used to size damage or quantify severity accurately. Its primary contribution lies in enhancing images, thereby facilitating better visualization and potentially deeper interpretation of damage indicators such as cracks, voids, or changes in wood density. Tomographic reconstructions offer the potential to produce actual images of the interior of wood in the form of density or velocity maps. However, many measurements, ideally from all sides of a member, are required for this imaging technique. Additionally, tomographic reconstruction algorithms are complex and computationally expensive.

Differentiating between various types of defects remains a significant limitation for both methods in wood inspection, even when imaging is employed. Both testing methods may have difficulty detecting voids smaller than a few millimeters, although their detection capabilities can vary depending on the damage’s relative size to the pulse’s wavelength. As a rule of thumb, if the wavelength is less than half the size of the feature of interest and a sufficient difference between the acoustic impedance or dielectric constant of the material and the feature exists, it may cause a reflection detectable by the equipment. The inverse relationship between frequency and penetration depth holds for both GPR and UST.

Future research should focus on developing advanced imaging algorithms such as tomographic reconstruction and full waveform inversion. These algorithms can account for wave propagation in complex, i.e., anisotropic and heterogeneous, media. Additionally, combining GPR and UST images using fusion algorithms may further improve damage detection, aid in quantifying damage size, and distinguish the nature of the damage. Sophisticated filtering algorithms, including adaptive median and Gaussian filtering, can be implemented to eliminate unwanted noise and enhance the visibility of relevant features in the images. Further exploration of the dependency of wave velocity with direction in the SAFT/TFM image intensity Equation (3) is warranted. Another aspect is the availability of testing equipment; while GPR and UST instruments that support imaging are commercially available for concrete applications, the same may not be said for timber. A concentrated effort is essential in researching and developing instruments and hardware tailored specifically for wood applications. This focus would ensure more accurate measurements, providing dependable data for precise image reconstruction. Finally, simulations, alongside machine learning and statistical analysis, can be crucial in this developmental process. This entails training models and labeling measurements across various wood types and defect scenarios, potentially automating defect detection and characterization across different wood types.

## Figures and Tables

**Table 1:** Dielectric constants of Euroamerican hybrid Poplar, Alder, Oriental beech, and Spruce woods at different moisture contents and structural directions [21]. The last row of data (for Spruce) is based on the authors’ measurements on a Spruce CLT specimen (see Appendix A, Figure A1 for data).

Frequency (GHz)	Wood Species	Dielectric Properties	Moisture Content (%)			
			0	15	28	33
2.45	Poplar	$\epsilon_{l}^{\prime }$	1.7	2.88	3.93	-
		$\epsilon_{⊥}^{\prime }$	1.48	2.06	2.85	-
	Alder	$\epsilon_{l}^{\prime }$	1.88	3.1	4.33	-
		$\epsilon_{⊥}^{\prime }$	1.63	2.34	3.4	-
	Oriental beech	$\epsilon_{l}^{\prime }$	2.26	3.38	4.77	-
		$\epsilon_{⊥}^{\prime }$	1.93	3.04	4.38	-
2.7	Spruce	$\epsilon_{r}$	-	-	-	2.18

$\epsilon_{l}^{\prime }$ is dielectric constant in the longitudinal direction, and $\epsilon_{⊥}^{\prime }$ is dielectric constant in the transverse direction. $\epsilon_{r}$ is measured through the thickness of the mass timber panel.

**Table 2:** Properties of different types of sound wood [8,30].

Wood Species	Density (g/cm^3^)	Modulus of Elasticity (kg/mm^2^)	Longitudinal Stress Wave Velocity Parallel to Grain (m/s)	Longitudinal Stress Wave Velocity Perpendicular to Grain (m/s)
Ash, White	0.638	1249	3968–5076	-
Beech	0.655	1180	-	1670
Birch, Yellow	0.668	1400	4348–5748	1399–1480
Cherry, Black	0.534	1046	4831–5435	1451–1613
Pine	0.3–0.7	850–1450	5000–5883	1066–1146
Spruce	0.4–0.7	1000–1070	5883	931–1310
Oak, Live	0.977	1381	-	627–1631
Oak, Red	0.657	1274	3311–5882	1548–1754
Oak, White	0.710	1251	-	1258
Maple, Sugar	0.676	1290	3906–5155	-

**Table 3:** Summary of commonly used non-destructive and semi-destructive testing methods for timber.

Name	Physical Principle	Working Principle (Equipment Used)	Applications	Use	Use as an Imaging Method	Available Codes/Standards
**Non-destructive Techniques**						
Sounding	Sound waves	- Sound wave generator (hammer)	Identifying degraded areas, cracks, voids, thickness	Both lab and field settings for both live and processed timber	×	
IR	Infrared thermal radiation	- Internal heat flow artificially or using natural sources - Infrared cameras and sensors	Identifying cracks, voids, mechanical failures, impurities	Both lab and field settings for both live and processed timber	✓	-ASTM D4788-03 [76]
X-ray	Electromagnetic radiation	- X-ray generator - Digital detectors - Phosphor-layered imaging plate	Identifying internal structure, corrosion, defects, cracks	Typically, lab-based for safety	✓	-ASTM E2767-21 [77]
UST	Sound waves (ultrasonic)	- Ultrasonic transducers and receivers - Signal generator - Amplifier - Data acquisition system	Detecting internal defects, measuring material thickness, assessing material properties, cracks, delamination, corrosion, structural integrity	Field and lab, live timber, processed timber	✓	-ISO 16810 [78], -ISO 16827 [79], -ASTM E2663-14 [80]
GPR	Electromagnetic waves	- Transmitting and receiving antenna - Pulse generator - Data acquisition unit	Detecting internal defects, decay, void, cracks, moisture	Field and lab; can be applied to both live and processed timber	✓	-ASTM D6432-19 [81]
ER	Variation of electrical conductivity of wood	- Using electrodes inserted into timber - Measuring the resistance between electrodes	Detecting moisture and decay in timber	Field and lab, processed and live timber	✓	
**Semi-destructive Techniques**						
DR	Mechanical friction	- Drilling equipment	Assessing the density and hardness of different wood species, quantify deterioration	Usable in the field, but not a standard method for assessing timber quality	×	

**Figure A1:**
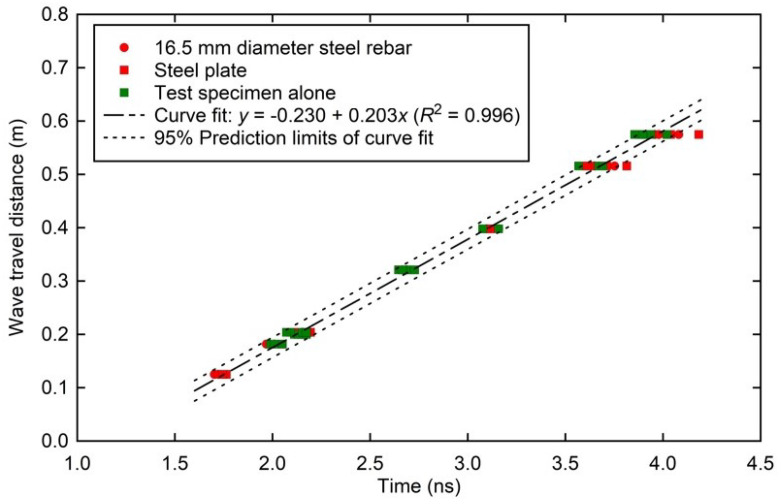
Relationship between time and wave travel distance for Spruce timber CLT specimen. Wave travel distances were measured experimentally by recording the time for waves to traverse specific distances. The data were then curve-fitted to establish the relationship between time and wave travel distance (authors’ work).

## Data Availability

Data generated by the authors are available from the corresponding author, N.P., upon reasonable request.
